# The auxin response factor gene family in allopolyploid *Brassica napus*

**DOI:** 10.1371/journal.pone.0214885

**Published:** 2019-04-08

**Authors:** Jing Wen, Pengcheng Guo, Yunzhuo Ke, Mingming Liu, Pengfeng Li, Yunwen Wu, Feng Ran, Mangmang Wang, Jiana Li, Hai Du

**Affiliations:** 1 College of Agronomy and Biotechnology, Chongqing Engineering Research Center for Rapeseed, Southwest University, Chongqing, China; 2 Academy of Agricultural Sciences, Southwest University, Chongqing, China; Huazhong University of Science and Technology, CHINA

## Abstract

Auxin response factor (ARF) is a member of the plant-specific B3 DNA binding superfamily. Here, we report the results of a comprehensive analysis of ARF genes in allotetraploid *Brassica napus* (2n = 38, AACC). Sixty-seven ARF genes were identified in *B*. *napus* (*BnARFs*) and divided into four subfamilies (I–IV). Sixty-one *BnARFs* were distributed on all chromosomes except C02; the remaining were on Ann and Cnn. The full length of the BnARF proteins was highly conserved especially within each subfamily with all members sharing the N-terminal DNA binding domain (DBD) and the middle region (MR), and most contained the C-terminal dimerization domain (PBI). Twenty-one members had a glutamine-rich MR that may be an activator and the remaining were repressors. Accordingly, the intron patterns are highly conserved in each subfamily or clade, especially in DBD and PBI domains. Several members in subfamily III are potential targets for miR167. Many putative cis-elements involved in phytohormones, light signaling responses, and biotic and abiotic stress were identified in *BnARF* promoters, implying their possible roles. Most ARF proteins are likely to interact with auxin/indole-3-acetic acid (Aux/IAA) -related proteins, and members from different subfamilies generally shared many common interaction proteins. Whole genome-wide duplication (WGD) by hybridization between *Brassica rapa* and *Brassica oleracea* and segmental duplication led to gene expansion. Gene loss following WGD is biased with the A_n_-subgenome retaining more ancestral genes than the C_n_-subgenome. *BnARFs* have wide expression profiles across vegetative and reproductive organs during different developmental stages. No obvious expression bias was observed between A_n_- and C_n_-subgenomes. Most synteny-pair genes had similar expression patterns, indicating their functional redundancy. *BnARFs* were sensitive to exogenous IAA and 6-BA treatments especially subfamily III. The present study provides insights into the distribution, phylogeny, and evolution of ARF gene family.

## Introduction

Auxin response factors (ARF) are a group of transcription factors generally consisting of an N-terminal DNA-binding domain (DBD), a middle region (MR), and a C-terminal dimerization domain (PBI) [1,2]. The DBD was further classified into three distinct structural domains based on crystal structure analysis: a B3 domain showing similarity to the DNA-contacting domain of bacterial endonucleases, two dimerization domains (DD1 and DD2) allowing ARF dimerization, and a Tudor-like ancillary domain (AD) of unknown function which might be involved in an interaction with the DD [3]. The DBD of ARFs fulfills a critical role for a transcription factor: recognition of a DNA motif, called the auxin-responsive element (AuxRE). The MR enriched in glutamine (Q), serine (S), and leucine (L) residues is identified as the activation domain, while those enriched in S, proline (P), L, and glycine (G) residues are identified as repression domains [1]. The PBI consists of domain III and domain IV. The N-terminal III region consists of an antiparallel β-sheet (β1–β2) and α1. The C-terminal IV region contains a second antiparallel β-sheet (β3–β5) and two α-helices (α2 and α3). The PBI domain is the most optional one and is similar to motifs III and IV of the Aux/IAA protein family [4], which is not present in certain ARF proteins such as AtARF3 and AtARF17 [5,6]. The auxin response is not fully dependent on PBI [7]. Transient expression assays and domain swaps have demonstrated that each of the three domains can act in isolation [2].

After the first ARF gene (*AtARF1*) was cloned from *Arabidopsis* [8], a large gene family containing 23 ARFs has been isolated, also from *Arabidopsis* [9,10]. Many homologous genes that play important roles in diverse biological processes in plants, such as controlling plant growth, and developmental and physiological processes in the whole life cycle of plants have been functionally characterized [11–13]. For instance, *AtARF17* plays an essential role in anther development and pollen formation in *Arabidopsis* [14]; *AtARF2-4* and *AtARF5* are essential for female and male gametophyte development in *Arabidopsis* [15]; *SlARF3* plays a key role in the formation of epidermal cells and trichomes in tomato [16]. Unlike most plant transcription factors, ARFs can act as both activators and repressors. ARF activators bind to TGTCTC auxin response elements (AuxRE) in promoters of auxin response genes to mediate auxin-dependent transcriptional regulation [8]. ARF repressors may squelch the transcriptional activity of activator ARFs either by heterodimerizing with them or through competition for DNA binding sites [17]. The PBI is not required for DNA-binding or activation but facilitates the formation of ARF-ARF, ARF-Aux/IAA, and Aux/IAA-Aux/IAA homo- and hetero-oligomers [18–20]. Owing to the increasing amount of available plant genome data, the ARF gene family were identified in some species at genome-wide level. For example, 23 genes were identified in *Arabidopsis* (*AtARFs*), 22 genes in tomato, and 28 genes in chickpea [10,21,22]. These studies provide the fundamental information needed for the functional analysis of ARF genes. However, only one gene, named *ARF18*, has been functionally analyzed in *Brassica napus* which affects seed weight and silique length [23]. It is clear that the genome-wide identification and functional analysis of this gene family in *B*. *napus* will provide fundamental information for further functional assays of ARF genes in this species.

In the present study, we carried out systematic identification and phylogenetic analysis of the ARF genes in the *B*. *napus* genome. Gene structure and protein motif analysis provided an additional criterion for our classification based on the phylogenetic tree. Cis-element analysis of the promoters of candidates indicated that they were involved in light signaling response. The whole genome-wide duplication (WGD) and segmental duplication mainly contributed to the rapid expansion of this gene family in *B*. *napus*, whereas no tandem duplication was observed. Expression profile analysis showed that *B*. *napus* ARF genes have wide expression profiles across the vegetative and reproductive organs during different developmental stages, and that some members were extra-hormone mediated. This study provides valuable information regarding the evolution, radiation, and functional diversification of the ARF gene family in *B*. *napus*, which will facilitate further analysis of their functional traits.

## Material and methods

### Identification of ARF family genes and chromosome location in *B*. *napus*

The sequences of 23 *Arabidopsis* ARF genes were downloaded from the TAIR *Arabidopsis* genome (http://www.arabidopsis.org/) [24]. We performed a BLASTP search of the GNOSCOPE genome database (http://www.genoscope.cns.fr/brassicanapus/) using the full protein sequences of *Arabidopsis* ARF genes as queries. To verify the reliability of our results, the protein functional and structural domains were predicted with PROSITE profiling (http://www.expasy.org/tools/scanprosite/) [25] to confirm that each protein had the B3 domain. We acquired *B*. *oleracea* and *B*. *rapa* ARF protein sequences from Phytozome (https://phytozome.jgi.doe.gov/pz/portal.html) and BRAD (http://brassicadb.org/brad/index.php) by the same method.

The biochemical properties of the candidates were predicted by ExPASy [26]. Subcellular location was investigated using Cell-PLoc [27]. The gene locations of candidates were extracted from the GNOSCOPE genome database. MapChart v2.3 software was used to view the chromosome locations of candidates [28]. The physical and chemical characteristics of the candidate BnARFs proteins were predicted by Protparam tool (http://web.expasy.org/) [29], received the theoretical pI/Mw for the BnARFs sequence.

### Phylogenetic and synteny analysis of *B*. *napus* ARF proteins

To examine the evolutionary history of the *B*. *napus* ARF gene family, we constructed a neighbor-joining (NJ) tree based on the multiple alignment of the ARF domains using MEGA 5.1 [30]. To determine the statistical reliability, we applied bootstrap analysis with 1000 replicates, and the main parameters were p-distance and pairwise deletion. The tree file was viewed and edited using FigTree v1.3.1 (http://tree.bio.ed.ac.uk/software/figtree/). Using the same method, we constructed a NJ tree of *B*. *napus*, *B*. *rapa*, *B*. *olerace*a, and *Arabidopsis* ARF proteins. Synteny relationships of ARF genes from *Arabidopsis*, *B*. *napus*, *B*. *oleracea*, and *B*. *rapa* were acquired from CoGe (https://genomevolution.org/coge/).

### Protein sequence analysis and construction of the protein interaction network

To reveal the sequence features of *BnARF* proteins, we performed a multiple alignment analysis of the full protein sequences with MAFFT version 7 using the default parameters (https://mafft.cbrc.jp/alignment/server/). To obtain optimized alignments, the deduced amino acid sequences were adjusted manually in MEGA 5.0 with default parameters. MEME version 4.11.1 [31] was used to identify potential protein motifs using the following parameters: distribution of motifs, maximum number of motifs, 20; minimum width of motif, six; maximum width of motif, 50. Only motifs with an e-value ≤ 1e-10 were retained for further analysis. The protein–protein interactions (PPI) network of *AtARFs* was acquired based on publicly available data in STRING [32]. Then we predicted the PPI network of *BnARFs* based on orthology analysis with *AtARFs* and visualized with Cytoscape version 3.4.0 [33].

### Analyses of gene structure, miRNAs, and cis-elements

The intron insertion pattern (including the distribution, position, and phases) and DBD and PBI encoding regions in the *B*. *napus* and *Arabidopsis* ARF genes were analyzed and viewed by Gene Structure Display Server software [34] (http://gsds.cbi.pku.edu.cn/). Plant small RNA targeted gene prediction was performed by psRNATarget (http://plantgrn.noble.org/psRNATarget/analysis/) [35]. To identify the putative cis-elements, the 1.5-kb upstream DNA sequences of all *B*. *napus* ARF genes were analyzed by the online PlantCARE web tool (http://bioinformatics.psb. ugent.be/webtools/plantcare/html/).

### Expression analysis of *B*. *napus* ARF genes at different developmental stages and under hormone treatments

The temporal and spatial expression patterns of candidate *BnARFs* were analyzed using the RNA-seq data (BioProject ID: PRJNA358784) from 50 different roots, stems, leaves, flowers, seeds, and siliques tissues of the *B*. *napus* cultivar ‘Zhongshuang 11’ (ZS11) at germination, seedling, budding, initial flowering, and full-bloom stages. The expression information of candidates in seedling roots of ZS11 under five extra hormone inductions (e.g, auxin, gibberellin, cytokinin, abscisic acid, and ethylene) were collected for analysis. The data were analyzed by Cluster 3.0 [36] and the heatmap was drawn using Java Treeview software [37].

For qRT-PCR analysis, seeds of ZS11 were obtained from the College of Agriculture and Biotechnology, Southwest University, Chongqing, China and germinated on Petri dishes. At the five-leaf stage, the seedlings were treated with Hoagland's liquid medium, which contained phytohormones (75 μM 6-benzyladenine (6-BA) and 10 μM Indole-3-acetic acid (IAA)) and grown in an artificial climate chamber at 25°C under a 14-h/10-h (day/night) photoperiod. Root tissues were harvested 0, 1, 3, 6, 12, and 24 h after treatment, and then were immediately frozen in liquid nitrogen and stored at −80°C for RNA isolation.

The extraction of total RNA from root samples and subsequent cDNA synthesis were performed as described previously [38]. Total RNA was extracted using Eastep total RNA Extraction kit, according to the manufacturer’s instructions, and treated with DNase I (Promega, USA). First-strand cDNA synthesis was performed using an oligo (dT) primer (S1 Table) and 2 μg of total RNA in a 20-μl reaction volume with the M-MuLV RT kit (Takara Biotechnology, Japan), according to the manufacturer’s instructions. The real-time PCR thermocycling parameters were as follows: initial denaturation for 3 min at 95°C, followed by 45 cycles of denaturation at 95°C for 15 s and then annealing at 58°C for 20 s. Fluorescence was measured after the extension step using the CFX Connect Real-Time System (Bio-Rad). After the thermocycling reaction, the melting step was performed from 65°C to 95°C, with increments of 0.5°C each 0.05 s. Each PCR was replicated three times for verification.

## Results

### Sequence identification and chromosomal locations of *B*. *napus* ARF genes

A total of 67 *BnARFs* were identified in the *B*. *napus* genome, consisting of 62 typical ARF genes with a complete open reading frame (ORF) and five putative genes with short fragment deletions in the BDs (*BnaC03g57150D*, *BnaAnng13910D*, *BnaC09g52440D*, *BnaC09g23080D*, and *BnaA06g14090D*). This is the largest ARF gene family reported to date (Table 1). Finally, the 67 candidate *BnARFs* were classified and relevant nomenclature was applied according to their positions on *B*. *napus* chromosomes.

**Table 1:** Features of the 67 BnARF genes identified in *B*. *napus*.

Gene Name	Gene ID	mRNA size	Protein size	PI	MW(kDa)	Subcellular localization	Domain
BnARF01	BnaA01g06910D	2186	611	8.17	67650.95	Nucleus	DBD, PBI
BnARF02	BnaA01g13580D	2391	604	6.08	68577.77	Nucleus	DBD, PBI
BnARF03	BnaA01g35830D	2365	661	6.16	73467.60	Nucleus	DBD, PBI
BnARF04	BnaA02g05070D	3342	1113	6.43	123130.86	Nucleus	DBD, PBI
BnARF05	BnaA03g46370D	2503	625	6.19	70589.72	Nucleus	DBD, PBI
BnARF06	BnaA03g49980D	2647	830	8.74	92666.31	Nucleus	DBD, PBI
BnARF07	BnaA04g07950D	2781	819	5.94	90457.58	Nucleus	DBD, PBI
BnARF08	BnaA04g19890D	1975	594	6.63	65578.16	Nucleus	DBD
BnARF09	BnaA05g01310D	1955	584	6.53	65836.87	Nucleus	DBD, PBI
BnARF10	BnaA05g09790D	2096	602	6.38	66005.85	Nucleus	DBD
BnARF11	BnaA05g14370D	1085	302	9.67	34384.53	Nucleus	DBD
BnARF12	BnaA06g13320D	3525	1049	6.22	116148.44	Nucleus	DBD, PBI
BnARF13	BnaA06g14040D	2820	876	5.75	96924.97	Nucleus	DBD, PBI
BnARF14	BnaA06g14090D	856	248	7.15	28404.65	Nucleus	DBD
BnARF15	BnaA06g17250D	1665	554	6.54	62691.42	Nucleus	DBD, PBI
BnARF16	BnaA06g21460D	3818	851	6.35	94595.98	Nucleus	DBD, PBI
BnARF17	BnaA07g11660D	2892	865	5.85	95983.25	Nucleus	DBD, PBI
BnARF18	BnaA07g13830D	2350	685	7.61	75887.91	Nucleus	DBD, PBI
BnARF19	BnaA07g20790D	2009	545	5.09	59768.72	Mitochondrion Nucleus	DBD
BnARF20	BnaA07g25390D	2952	781	6.00	87343.73	Nucleus	DBD, PBI
BnARF21	BnaA08g17390D	2746	852	6.03	94105.51	Nucleus	DBD, PBI
BnARF22	BnaA08g22150D	3228	1020	6.52	112464.81	Nucleus	DBD, PBI
BnARF23	BnaA08g31250D	2995	846	5.55	93815.67	Nucleus	DBD, PBI
BnARF24	BnaA09g01790D	2169	547	6.18	62552.18	Nucleus	DBD, PBI
BnARF25	BnaA09g05840D	3060	877	6.15	97548.11	Nucleus	DBD, PBI
BnARF26	BnaA09g15480D	1653	550	5.98	62495.48	Nucleus	DBD, PBI
BnARF27	BnaA09g26170D	2869	873	5.85	96064.68	Nucleus	DBD, PBI
BnARF28	BnaA10g13260D	2433	757	6.35	83793.20	Nucleus	DBD, PBI
BnARF29	BnaA10g14760D	3312	1040	6.40	114043.61	Nucleus	DBD, PBI
BnARF30	BnaAnng09990D	1674	557	6.48	63649.50	Nucleus	DBD, PBI
BnARF31	BnaAnng13910D	1699	540	6.19	61368.92	Nucleus	DBD, PBI
BnARF32	BnaAnng25060D	1596	531	7.61	60771.14	Nucleus	DBD, PBI
BnARF33	BnaC01g08330D	2150	617	7.60	68183.46	Nucleus	DBD, PBI
BnARF34	BnaC01g15800D	1803	600	6.93	68209.64	Nucleus	DBD, PBI
BnARF35	BnaC01g28340D	1986	661	6.03	73454.56	Nucleus	DBD, PBI
BnARF36	BnaC03g25860D	1782	545	6.31	61351.97	Nucleus	DBD, PBI
BnARF37	BnaC03g52090D	3745	850	6.29	124917.51	Nucleus	DBD, PBI
BnARF38	BnaC03g57090D	1692	563	6.48	64146.99	Nucleus	DBD, PBI
BnARF39	BnaC03g57150D	1953	650	7.05	73465.62	Nucleus	DBD, PBI
BnARF40	BnaC03g59640D	2544	847	6.06	93443.86	Nucleus	DBD, PBI
BnARF41	BnaC04g15900D	2392	680	6.73	75551.51	Nucleus	DBD, PBI
BnARF42	BnaC04g18710D	1860	619	6.21	69275.00	Nucleus	DBD, PBI
BnARF43	BnaC04g53900D	1704	567	6.71	64607.67	Nucleus	DBD, PBI
BnARF44	BnaC05g14880D	3690	1058	6.30	117201.60	Nucleus	DBD, PBI
BnARF45	BnaC05g15390D	3223	879	5.84	97130.35	Nucleus	DBD, PBI
BnARF46	BnaC05g23210D	2860	870	5.86	96005.28	Nucleus	DBD, PBI
BnARF47	BnaC06g20640D	1882	544	5.26	59855.88	Mitochondrion Nucleus	DBD
BnARF48	BnaC06g27170D	2930	779	6.00	87278.74	Nucleus	DBD, PBI
BnARF49	BnaC06g38360D	1910	530	5.26	57804.61	Mitochondrion Nucleus	DBD
BnARF50	BnaC07g17570D	1113	370	8.54	43009.48	Nucleus	DBD
BnARF51	BnaC07g17860D	1674	557	6.46	63769.66	Nucleus	DBD, PBI
BnARF52	BnaC07g38640D	2306	625	6.11	70588.75	Nucleus	DBD, PBI
BnARF53	BnaC07g42330D	2686	844	7.80	94366.49	Chloroplast Nucleus	DBD, PBI
BnARF54	BnaC07g48490D	3224	861	5.83	95407.62	Nucleus	DBD, PBI
BnARF55	BnaC08g18670D	3063	1020	6.20	113085.42	Nucleus	DBD, PBI
BnARF56	BnaC08g19040D	3840	846	5.60	93764.61	Nucleus	DBD, PBI
BnARF57	BnaC09g00990D	1638	545	6.30	62375.96	Nucleus	DBD, PBI
BnARF58	BnaC09g05450D	3069	828	6.30	92424.42	Nucleus	DBD, PBI
BnARF59	BnaC09g23080D	2010	669	7.35	75501.91	Nucleus	DBD, PBI
BnARF60	BnaC09g23520D	1881	626	7.96	71792.28	Nucleus	DBD, PBI
BnARF61	BnaC09g35740D	2411	759	6.39	83959.26	Nucleus	DBD, PBI
BnARF62	BnaC09g37130D	3870	1042	5.75	110811.74	Nucleus	DBD, PBI
BnARF63	BnaC09g51490D	1506	501	6.37	57249.50	Nucleus	DBD, PBI
BnARF64	BnaC09g52440D	629	182	5.71	20758.57	Nucleus	DBD
BnARF65	BnaCnng05140D	2112	608	6.58	66687.63	Nucleus	DBD
BnARF66	BnaCnng25410D	2664	814	5.88	89645.76	Nucleus	DBD, PBI
BnARF67	BnaCnng54100D	3278	851	6.09	95522.78	Nucleus	DBD, PBI

In summary, the full-length coding sequences of *BnARFs* ranged from 1389 bp (*BnARF64*) to 7574 bp (*BnARF14*) with the deduced proteins varying from 182 (*BnARF64*) to 1058 (*BnARF44*) amino acids (Table 1). The predicted molecular weights of the 67 deduced BnARF proteins ranged from 20.76 kDa (*BnARF64*) to 124.92 kDa (*BnARF37*) and the isoelectric points (pIs) ranged from 5.26 (*BnARF47* and *BnARF49*) to 9.67 (*BnARF11*) (Table 1). As determined previously in other plant species [39,40], the wide range of pIs suggests that *B*. *napus* ARF proteins can function in different subcellular environments. Subcellular localization results showed that 63 *BnARFs* were localized in the nucleus, three in the mitochondrion nucleus, and one in the chloroplast nucleus. To further explore the evolutionary relationship in the *B*. *napus* ARF gene family after the formation of allopolyploids, 29 and 33 ARF genes were also obtained from the ancestors, *B*. *oleracea* and *B*. *rapa* genomes, respectively, using the same method. Detailed information regarding the candidates is listed in S2 Table.

**Fig 1:**
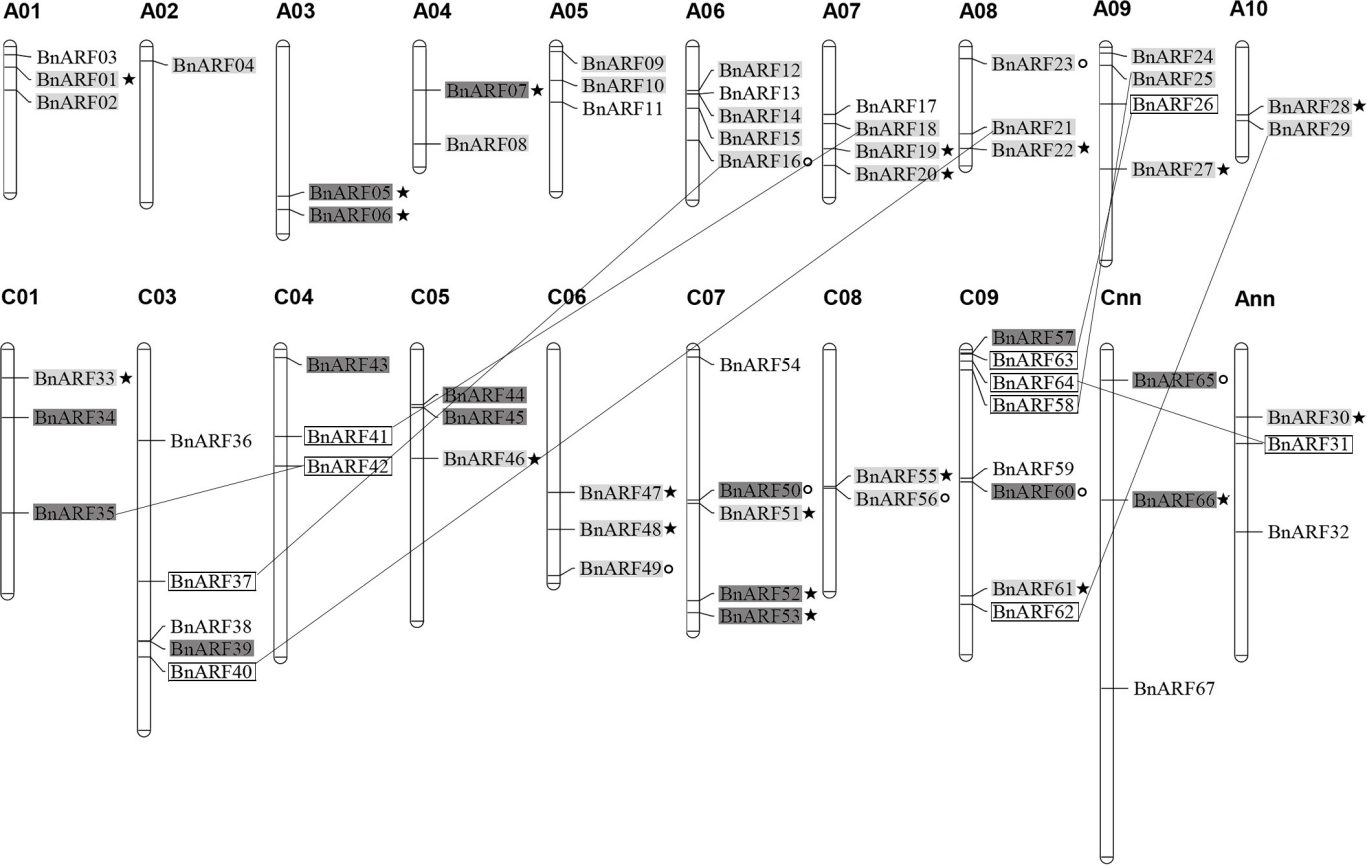
Distribution of *ARF* genes on *B*. *napus* chromosomes. In total, 67 *BnARF* genes were mapped to 18 chromosomes. The gray background represents the genes originating from *B*. *rapa*; the charcoal gray background represents the gene originating from *B*. *oleracea*; the black border represents the gene originating from segmental duplication, the black line represents the origin of the duplication; ★: homologous exchange (HE) event; ○: segmental exchange (SE) event.

Chromosomal location analyses revealed that 61 of the 67 *BnARFs* were mapped on 18 of the 19 chromosomes, i.e. three genes on A01, all except C02 (Fig 1). The remaining genes were in Ann and Cnn. The distribution of *BnARFs* in A_n_- and C_n_-subgenomes were nearly even, with 32 genes from the A_n_-subgenome and 35 from the C_n_-subgenome. However, the candidates on each chromosome were uneven. For example, C09 had the largest number (eight genes), while A02 had only one.

### Phylogenetic analysis and duplication of the *B*. *napus* ARF proteins

**Fig 2:**
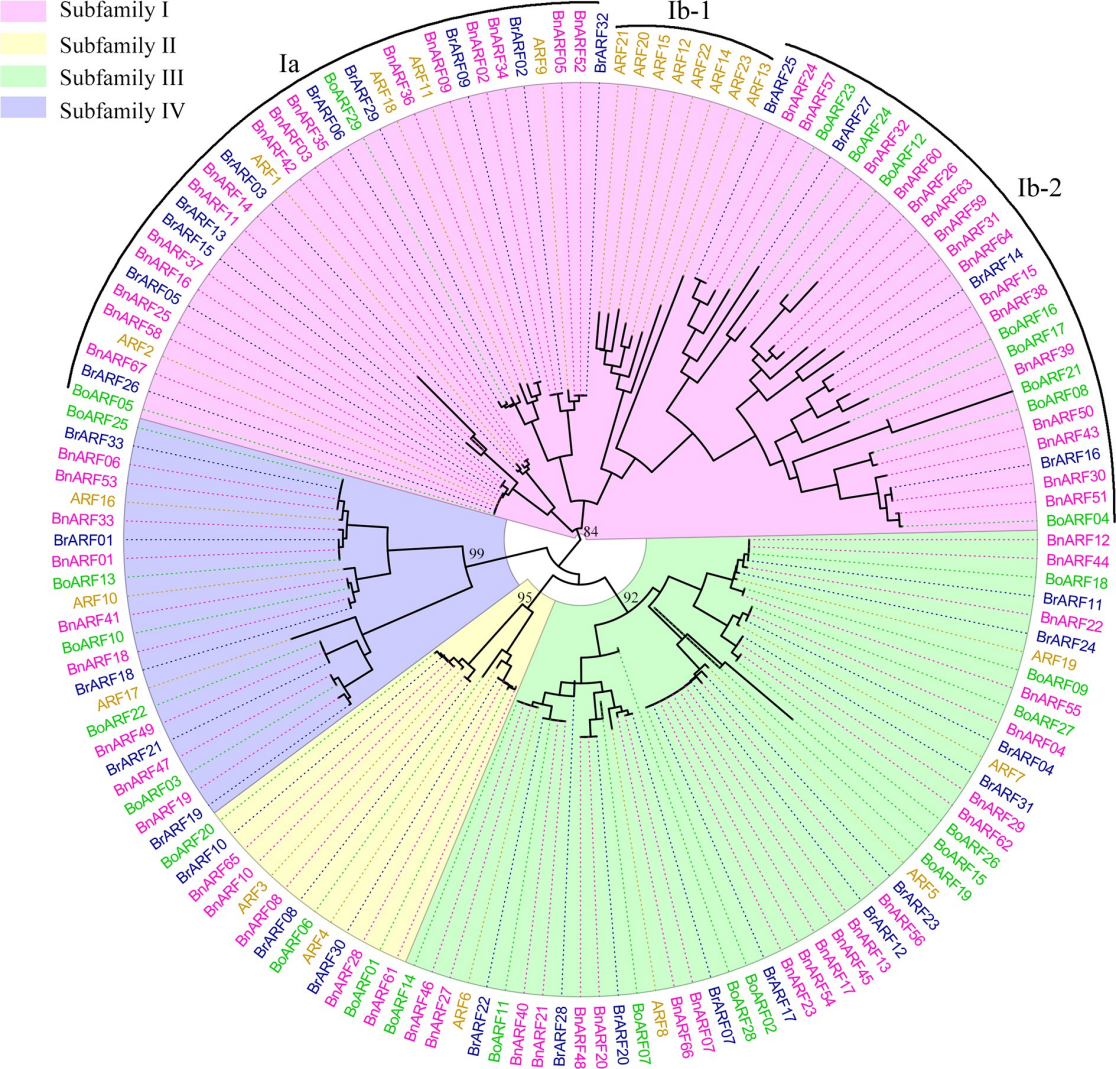
A neighbor-joining (NJ) tree of ARF proteins from *Arabidopsis*, *B*. *rapa*, *B*. *oleracea*, and *B*. *napus*. The NJ tree shows that the 23 *Arabidopsis*, 29 *Brassica oleracea*, 33 *Brassica rapa*, and 67 *B*. *napus* ARF proteins were clustered into four subfamilies (I–IV). Subfamilies I, II, III, and IV marked with red, yellow, green, and blue backgrounds, respectively. Genes in the four species labeled in different colors.

To explore the phylogenetic relationships among candidate ARF proteins, an unrooted NJ tree of the 152 ARF family members from *Arabidopsis* (23 genes), *B*. *oleracea* (29 genes), *B*. *rapa* (33 genes), and *B*. *napus* (67 genes) was constructed (Fig 2).

Similar to the results of previous studies, such as on *Arabidopsis* and rice [10,41], *Salvia miltiorrhiza* [42], and *Eucalyptus grandis* [43], all 152 ARF proteins were grouped into subfamilies I–IV containing 69, 13, 48, and 22 members, respectively. In the phylogenetic tree, members of each subfamily or clade were commonly found in the four species tested herein as sister-pairs. Except for the Ib clade in subfamily I, in which the ARF genes from *Arabidopsis* and the three Brassica species were clustered into two clearly separated subclades: Ib-1 and Ib-2 (Fig 2). Subclade Ib-1 was found only in *Arabidopsis* containing a fake gene (*AtARF23*) and seven tandem duplicated genes (*AtARF12–AtARF15* and *AtARF20–AtARF22* [10]); whereas, subclade Ib-2 contained *B*. *napus*, *B*. *rapa*, and *B*. *oleracea* genes. The Ib homologs were also not found in other plants, such as rice, maize, tomato, grape, or *Eucalyptus* [41,44–47], implying that this clade was formed of *Brassicaceae*-specific functions. Subfamily III had five *AtARFs*, 11 *B*. *rapa* ARF genes (*BrARFs*), 11 *B*. *oleracea* ARF genes (*BoARFs*), and 21 *BnARFs*. There were twice the number of *BrARFs* and *BoARFs* than *AtARFs* in this subfamily, whereas the number of *BnARFs* was likely to be the sum of *BrARF* and *BoARF* homologs. Similarly, subfamily IV had three *AtARFs*, five *BrARFs*, five *BoARFs*, and nine *BnARFs*, and there were around three times the number of *BnARFs* than *AtARFs*. However, this situation was different in the last two subfamilies. Subfamily I had 13 *AtARFs*, 14 *BrARFs*, 10 *BoARFs*, and 32 *BnARFs*, which indicates that the *BoARFs* and *BrARFs* in this subfamily tended to be lost compared to subfamilies III and IV. Similarly, subfamily II had two *AtARFs*, three *BrARFs*, three *BoARFs*, and five *BnARFs*, suggesting that the expansion of this subfamily in *Brassicaceae* was not as extensive as it was in the other subfamilies.

Based on syntenic analyses, a total of 59 *BnARFs* have syntenic relationships with *B*. *oleracea* or *B*. *rapa* homologs, resulting in 98 orthologous pairs among 32 *BrARFs* and 51 *BnARFs*, 63 orthologous-pairs among 22 *BoARFs* and 41 *BnARFs*, and 65 synteny-pairs within the *B*. *napus* genome (S3 Table). Among the 59 genes, 10 were derived from segmental duplication, the other 49 were derived from heterologous doubling; no tandem genes were observed (S4 Table). Moreover, 32 of the 49 heterologous doubling genes were derived from *B*. *rapa*, including 23 from the A_n_-subgenome (13 from orthology events, two from segmental exchange (SE) events, eight from homologous exchange (HE) events), and nine genes were transferred to the C_n_-subgenome by SE or HE events. Seventeen genes were derived from *B*. *oleracea* including 14 from the C_n_-subgenome and three were transferred to the A_n_-subgenome by HE events. These results indicated that gene loss following WGD is biased, with genes from *B*. *rapa* being retained and those from *B*. *oleracea* being readily lost. This confirms that the A_n_-subgenome replaced more of the C_n_-subgenome after WGD and featured more dominantly in each chromosome [48]. Given that *B*. *napus* evolved ~7500 years ago by hybridization (allopolyploidy) between *B*. *rapa* and *B*. *oleracea*, it was evident that WGD and segmental duplication contributed to the rapid expansion of ARF genes in *B*. *napus*, which may be independent of tandem duplication.

### Sequence features of *B*. *napus* ARF proteins

To explore the sequence features of candidate *BnARFs*, we further analyzed protein sequences of the N-terminal DBD, MR, and PBI. The DBDs of *BnARFs* generally consisted of three domains: DD (DD1 and DD2), B3, and AD [3]. The sequence features were quite different across these three domains. B3 and AD had conserved amino acid content and length while the DD was relative divergent. It was reported that some amino acid residues in the B3 domain play a vital role in the binding of ARF proteins to target DNA (*AtARF5*: H170, P218, R215, S165, S174, T227, and S230) [3].

**Fig 3:**
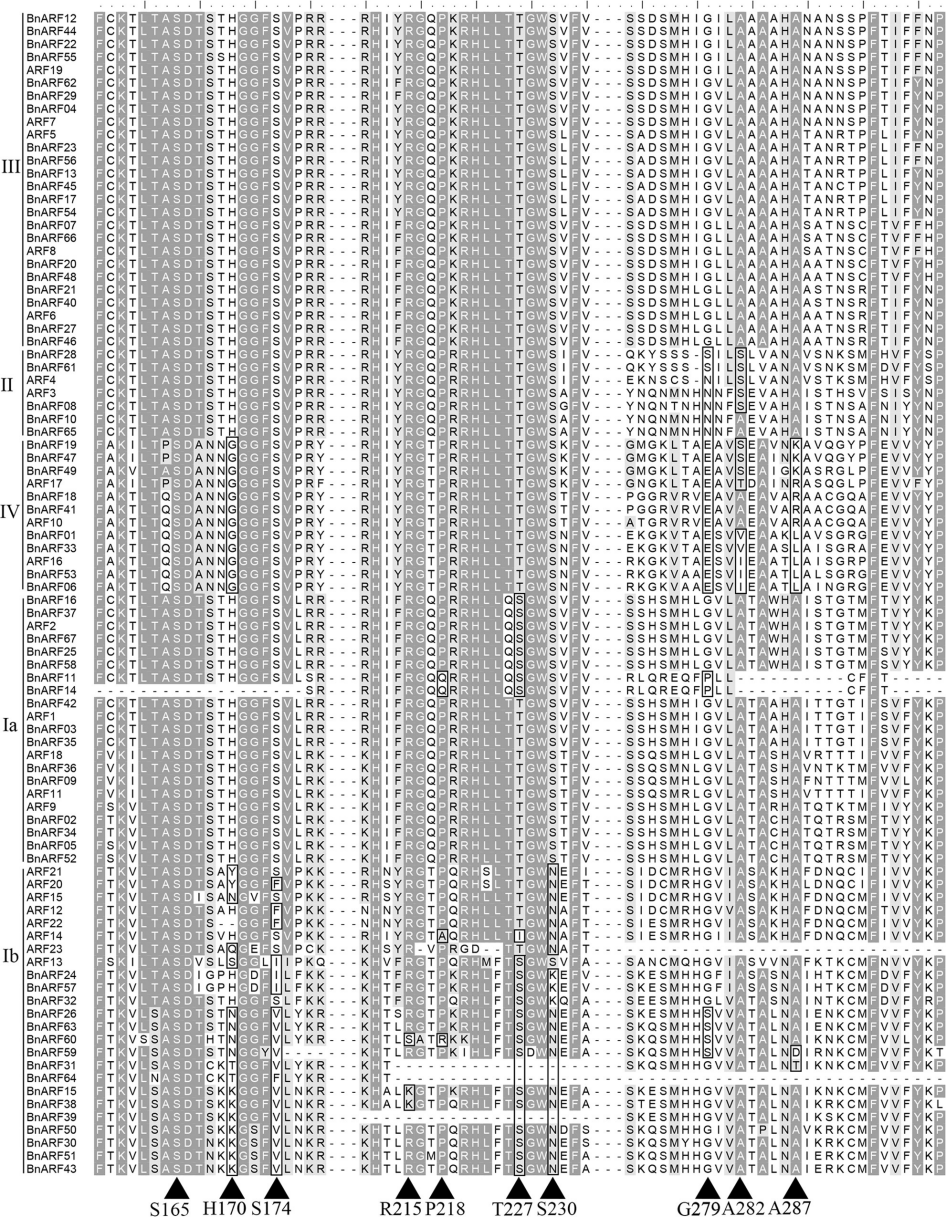
Key amino acid residues in B3 and DD domains. Triangle indicates the positions of key residues. The residues in the box represent substitution sites. The subfamily information is marked on the left.

In *Arabidopsis*, the mutant H170 residues of *AtARF5* lead to its DNA binding feature being significantly reduced. The mutants of P218 or R215 residues lead to its sequence-specific binding being lost; whereby S165, S174, T227, and S230 residues are involved in interactions with the DNA backbone, and the mutants of these four residues will reduce the binding ability of AuxRE [3]. It was reported that three residues (G279, A282 and A287) in the DD of *AtARF5* control the dimerization of the DD because substitutions at these sites resulted in functional differences [3]. In the present study, we found that the corresponding amino acids in these two domains were absolutely conserved in subfamily III and were highly conserved in subclade Ia (except the sequence inaccuracy of *BnARF11* and *BnARF14*), only the *AtARF2* and its homologs have one site substitution (from T227 to S227) (Fig 3). However, there are some conserved substitutions at these sites in other subfamilies as well, e.g, the H170 and G279 residues in the B3 and DD of subfamily IV were conserved in terms of being substituted into glycine (G) and glutamic acid (E), respectively. In subfamily II, the G279 and A282 residues were substituted into S279/N279 and S282, respectively. Moreover, there exists some non-conserved substitutions in subfamily IV (A282 and A287) and subclade Ib (H170, S174, T227, and S230) as well. Together, these results imply a possible role for the corresponding residues in functional diversification during evolution.

In the MR, the N-terminal is relatively conserved, but the C-terminal is not. According to the distribution and number of Q at its C-terminal, it could be divided into two groups. The first group which is rich in Q residues was suggested to contain activators, whereas those with fewer Q residues were suggested to be repressors. In *Arabidopsis*, AtARF5-8 and 19 function as transcriptional activators [1,2,5,49–51], the rest function as transcriptional repressors [2,5]. In *B*. *napus*, 21 *BnARFs* of subfamily III which were rich in Q residues (about twice that of others) were suggested to be activators, and the remaining 46 *BnARFs* in the other three subfamilies were suggested to be repressors (S1 Fig). In addition, both transcriptional activators and repressors of *BnARFs* have large amounts of S as well. Moreover, we found that *B*. *napus* transcriptional repressors have three- and two-times the amount of E and lysine (K) compared to transcriptional activators. This may also be important for gene functions.

**Fig 4:**
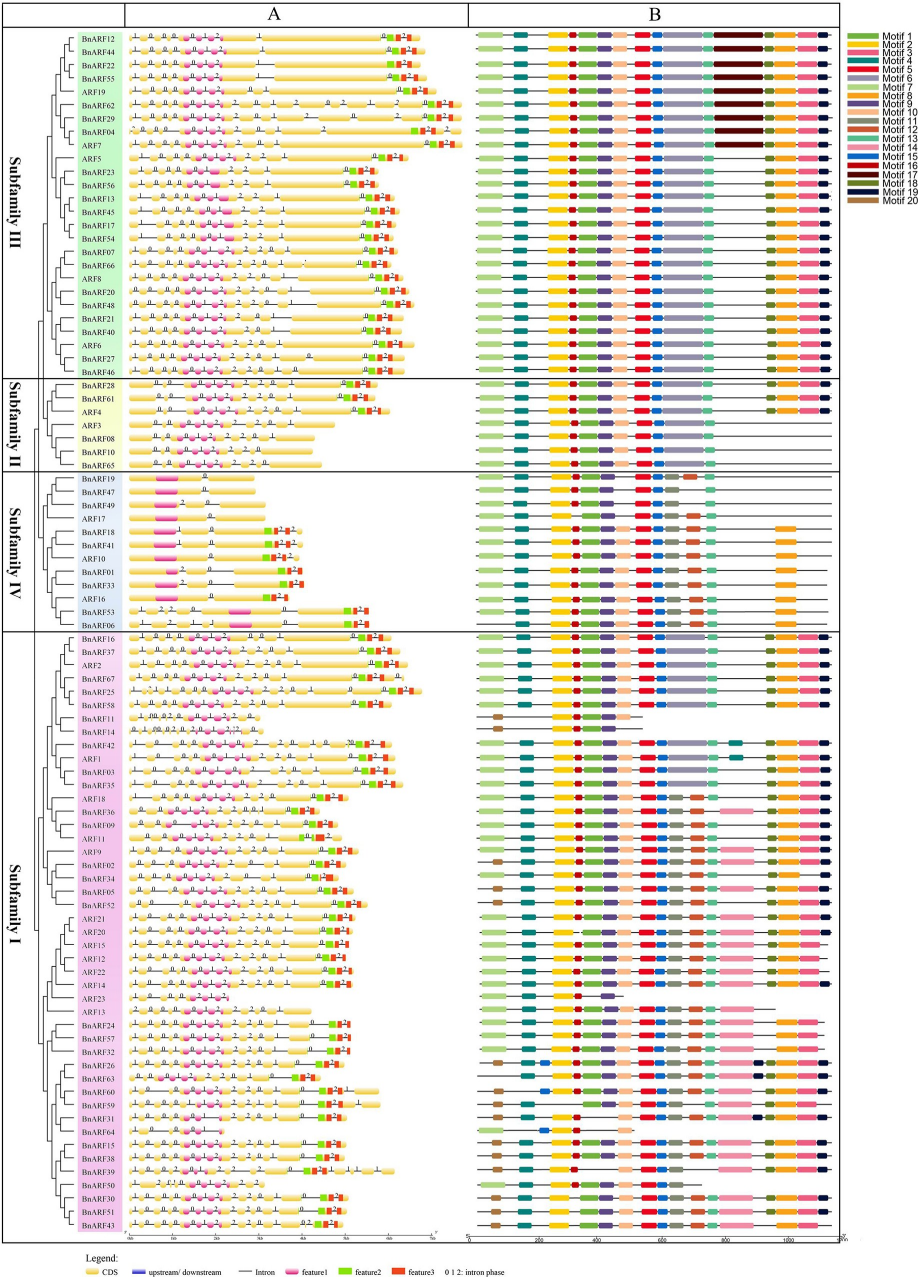
Gene structure and motif analysis of *B*. *napus* ARF genes. A: Gene structures of *BnARFs*; exon indicated by yellow boxes, B3 domain by fuchsia boxes, PBI domain by green (domain III) and red (domain IV) boxes, and the spaces between the colored boxes correspond to introns. B: The motifs identified in BnARF proteins by MEME analysis.

The PBI domain mediates interactions with Aux/IAA proteins [8]. We found that 10 *BnARFs* (8, 10, 65, 47, 49, 19, 11, 14, 64, and 50) lack the PBI domain (Fig 4). Among which, *BnARF8*, *10*, and *65* were the homologs of AtARF3, *BnARF47*, *49* and *19* were the homologs of *AtARF17*. As both *AtARF3* and *AtARF17* lack the PBI domain, it is suggested that homologs of these genes have conserved sequence features during evolution. *Arabidopsis* homologs and their homologs in *B*. *rapa* (*BrARF13*) have a conserved fragment deletion in the PBI domain, implying that the deletions in *BnARF11* and *BnARF14* originated from the same ancestor. While the fragment deletion of the PBI domain in *BnARF64* and *BnARF50* may be due to inaccurate sequencing or gene mutation, as their ancestor proteins were complete.

The MEME online software was further applied to identify motifs in the 67 *BnARFs* and 23 *AtARFs*. Altogether, 20 motifs were isolated (Fig 4). Among them, motifs 7, 20, 4, 2, 16, 1, 9, 10, 5, 15, 6, 11, 12, and 13 consisted of the DBD; motif 7, 20, and 4 consisted of the DD1; motifs 2, 16, 1, 9, and 10 consisted of the B3 domain; motifs 5 and 15 consisted of the DD2; motifs 6, 11, 12, and 13 consisted of the AD; motifs 17 and 14 consisted of the MR; while motif 18, 8, 3, and 19 consisted of the PBI domain. No other new motifs were observed in this study. This suggested that the structures of the ARF gene family are relatively conservative, especially within the same subfamily. Taken together, the homologous genes between *Arabidopsis* and *B*. *napus* commonly had the same sequence features, indicating functional conservation. The sequence features of subfamilies I, II, and III were similar, but those of subfamily IV were different.

### Gene structure analysis and small RNA predictions of *B*. *napus* ARFs

As the positions, lengths, and phases of introns were generally consistent with the phylogenetic relationships of a gene family [52], a comparison of the full-length cDNA and DNA sequences of each *BnARF* was performed to determine the numbers and positions of exons and introns. It was shown that there were many intron insertions in the coding regions of *BnARFs*, and the number of introns was different (from 1–17) across distinct subfamilies (Fig 4). For example, members in subfamily I generally had 13 intron insertion sites (except for a few genes with incomplete sequences). Members in subfamily II usually have eight or 11 introns; members in subfamily III have 10 or 12 introns, while those in subfamily IV have one–four introns (Fig 4). Interestingly, unlike other transcription factor gene families [52], the DNA-binding domain, intron insertion site, phase, and even the number of introns are highly conserved in *BnARFs* within a given subfamily or clade, which provides evidence supporting the reliability of our phylogenetic classification.

Among the three major domains (DBD, MR, and PBI) of ARF proteins, the intron patterns in the DBD and PBI domain were relatively more conserved than those in the MR (Fig 4). Moreover, the intron patterns in the DBD and PBI domain were the same across subfamilies I–III, but not in subfamily IV. For instance, nearly all the members in subfamilies I–III have three conserved intron insertion sites in B3 and two in the PBI domain. However, no introns were detected in B3 of subfamily IV, and only one conserved intron insertion site, which was the same as the second conserved intron insertion site in the PBI domain of the other subfamilies, was present in the PBI domain of some members. Therefore, although the number of intron insertions between subfamily IV and other subfamilies is different, the insertion site is conserved. However, the intron patterns in the MR were less conserved across different subfamilies, but it was relatively conserved within a given subfamily or clade.

Previous studies have revealed that several ARF genes are targets for small RNAs. For example, *AtARF6* and *AtARF8* in subfamily III are miR167 targets [53]; *AtARF10*, *AtARF16*, and *AtARF17* in subfamily IV are miR160 targets [54]. Accordingly, we found that all homologs of *AtARF6*, *AtARF8*, *AtARF10*, *AtARF16*, and *AtARF17* in *B*. *napus* may be potential targets for miR160 or miR167 (S5 Table). Moreover, the complementarity of the small RNA targets in subfamily IV is from 85–90%, while in subfamily III this is from 55–65% (S5 Table). This result suggests that target sequences of small RNAs have undergone differentiation and mutation in *B*. *napus* during evolution.

### Promoter region analysis of *B*. *napus* ARF genes

Transcription factors are known to bind to specific cis-elements and modulate the expression of target genes. Therefore, analysis of cis-elements in the upstream regulatory region of a gene can provides insights regarding the regulatory regime it follows. Therefore, we analyzed the 1.5 kb upstream sequence of all *BnARFs* (S2 Fig). In total, 106 cis-elements were identified in promoters of the 67 *BnARFs*, such as ARF binding sites, ethylene responsive elements, and drought responsive elements (ACCGAGA). Three putative auxin response cis-elements, TGA-box, TGA-element, and AuxRR-core were also identified, which are involved in 9, 9, and 27 *BnARFs*, respectively. Moreover, putative cis-elements involved in light signaling perception were observed in nearly all promoters of *BnARFs*, which may be the reason of the strong crosstalk between auxin and light signals [6,55]. Moreover, putative cis-elements involved in hormones, such as abscisic acid (ABA), gibberellin acid (GA), ethylene, and methyl jasmonic acid were found in a series of *BnARF* promoters. In addition, putative cis-elements associated with biotic and/or abiotic stress, such as dehydration, low temperature-, and heat stress-response elements were identified in most promoters of *BnARFs* (S6 Table). Overall, the evidence of diverse cis-elements in promoter regions of *BnARFs* suggested that *ARF* genes may be linkers between auxin response and other important processes, and thus, their regulatory networks should be focused on in future research [56,57].

### Interaction network of *B*. *napus* ARF genes

According to the visualization of the interaction relationship in STRING, 53 interaction proteins of 21 of the 23 *AtARFs* were obtained, including 26 Aux/IAA proteins, 11 ARF proteins, and 16 other proteins (such as TPR1/2/3, SWI3A/3B/3C and BIN etc.) (S6 Table). We observed that members of subfamily IV interacted with those of subfamily I, II, and III, while subfamily II could interact with subfamily III, implying that ARF proteins from different subfamilies commonly interact with each other. Moreover, *AtARF4* and *AtARF9* widely interacted with members of the other four subfamilies.

**Fig 5:**
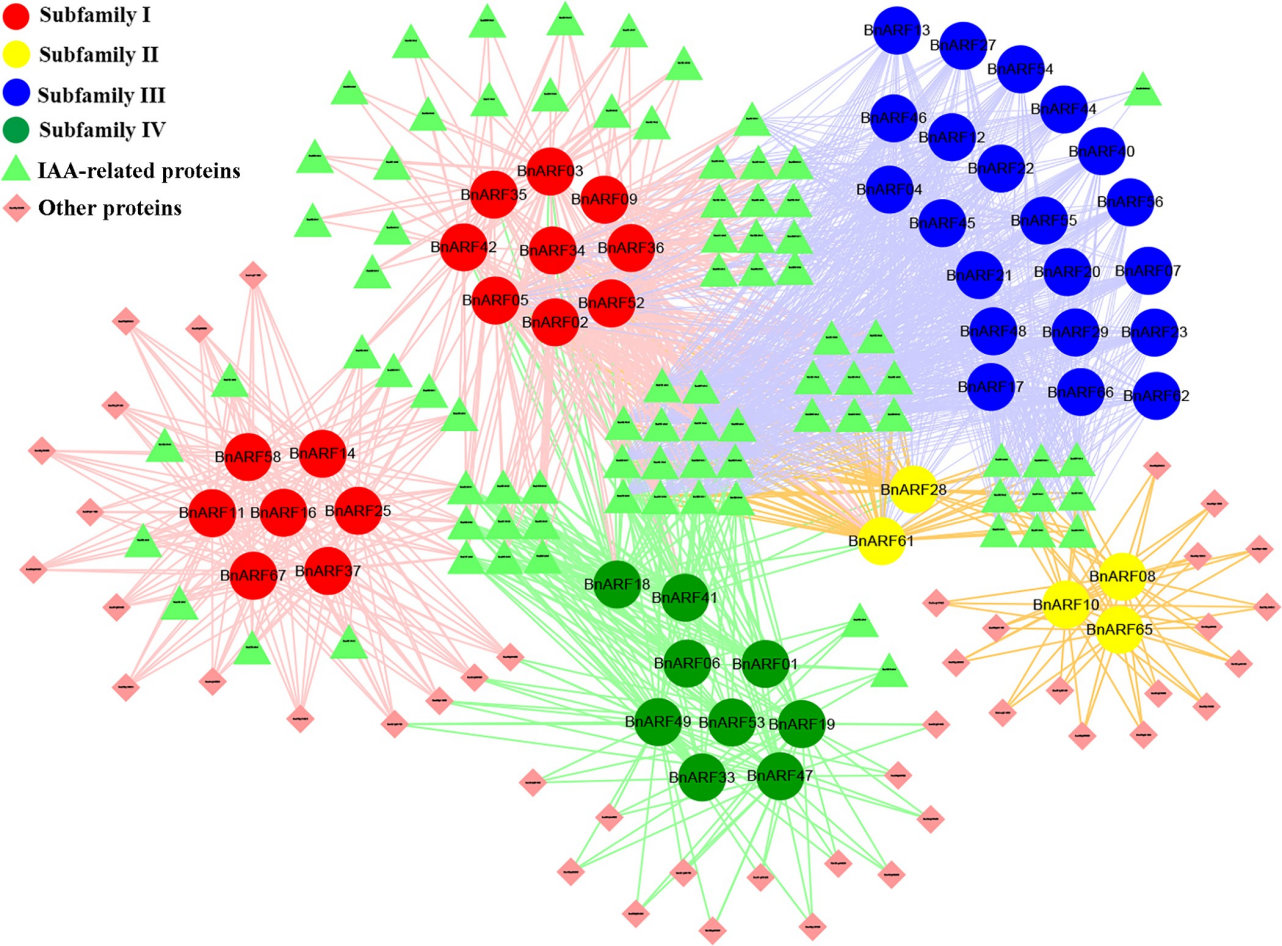
Interaction network of BnARF proteins in *B*. *napus*. The ARF proteins in the four subfamilies are represented by dots of different colors. Red: subfamily I; green: subfamily II; blue: subfamily III; yellow: subfamily IV. The Aux/IAA genes are represented by green triangles, and the other type of interaction proteins are represented by aquamarine blue quadrilaterals.

In *B*. *napus*, based on orthology analysis, 14 of the 21 *AtARFs* (except the genes belonging to the Ib-1 subclade) were found to have syntenic relationships with 51 *BnARFs* including 16 *BnARFs* from subfamily I, five from subfamily II, 21 from subfamily III, and nine from subfamily IV (Fig 5) (S6 Table). Furthermore, 49 of the 53 *Arabidopsis* interaction proteins have 169 homologs in *B*. *napus*. GO analysis showed that up to 96 of the 169 *B*. *napus* putative interaction proteins were IAA related proteins, 30 were ARF proteins, and 43 belonged to other proteins. Moreover, we found that members of subfamily I interacted with 70 IAA proteins, eight ARF proteins, and 15 other proteins; subfamily II interacted with 31 IAA proteins, four ARF proteins, and 17 other proteins; subfamily IV interacted with 25 IAA proteins, nine ARF proteins, and 17 other proteins, whereas subfamily III merely interacted with 45 IAA proteins and six ARF proteins. This evidence supports the functions of subfamily III, which interacts with IAA to regulate auxin response. Furthermore, we found that many interaction proteins were shared by different subfamilies, such as subfamilies I and III both interacting with 14 IAA proteins; subfamilies I and IV both interacting with nine IAA proteins and four other proteins; subfamilies I, II, and III all interacting with 17 IAA proteins. There were 14 IAA proteins that could interact with the proteins in all subfamilies simultaneously (Fig 5). Similar to the situation in *Arabidopsis*, the homologs of *AtARF4* (*BnARF61* and *BnARF28*) and *AtARF9* (*BnARF02*, *BnARF05*, *BnARF34*, and *BnARF52*) widely interacted with the *BnARFs* in the other four subfamilies.

Overall, our results showed that ARF proteins are likely to functions in interaction relationships, and that most interaction proteins are IAA-related proteins. The ARF proteins from different subfamilies generally shared many common interaction proteins and tended to interact with each other as well.

### Expression profiles of *B*. *napus* ARFs during different developmental stages

**Fig 6:**
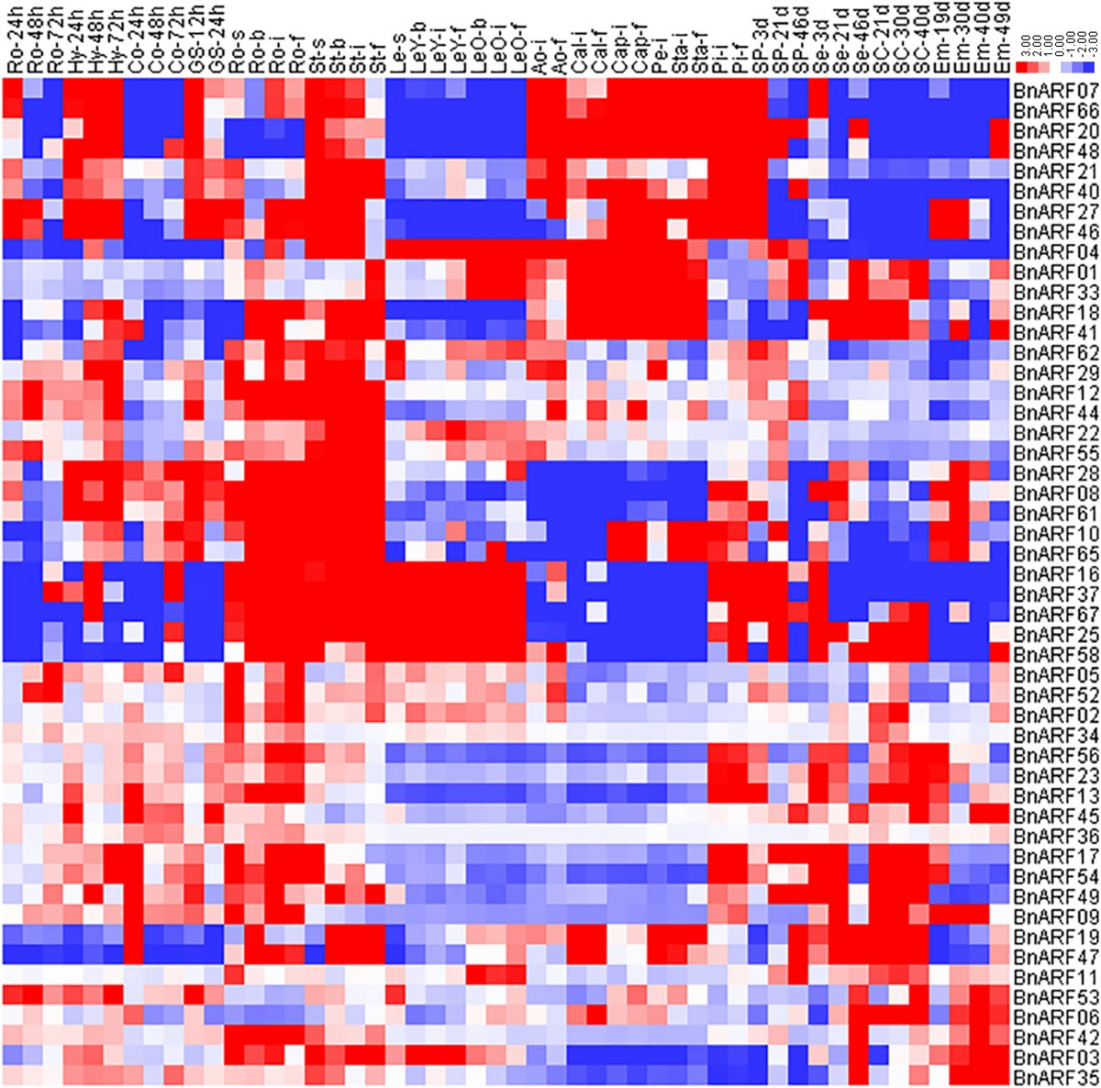
Expression profiles of *BnARFs* across different developmental stages and organs. Genes and their corresponding clades are presented on the right. Tissues used for expression analysis are indicated at the top of each column. GS, germinate seed; Hy, hypocotyl; Ao, anthocaulus; Ro, root; St, stem; Le, leaf; Cal, calyx; Cap, capillament; Pe, petal; Sta, stamen; Pi, pistil; SP, silique; Se, seed; SC, seed coat; Em, embryo; Co, cotyledon. s, seedling stage; b, bud stage; i, initial flowering stage; and f, full-bloom stage. The 24, 48, and 72 h indicate the time after seed germination. The 3, 19, 21, 30, 40, and 46 d indicate the days after pollination. The color bar represents log_2_ expression values (FPKM ≥ 1).

Gene expression patterns are generally related to gene functions; therefore, the expression of the 67 *BnARFs* in 50 tissues during different stages was investigated, based on the RNA-Seq data (Bio Project ID PRJNA358784). The genes with FPKM values of <1 were not included in the analysis. As shown in Fig 6, the candidate *BnARFs* have wide expression profiles across different vegetative and reproductive organs during different stages in the development of *B*. *napus*. In general, the expression patterns between different subfamilies and even different clades of a given subfamily were different but were very similar within the same clade. In subfamily I, the candidates were divided into two major clades: the genes in the Ib clade nearly had no expression value in all tissues investigated, whereas the genes in the Ia clade (16 *BnRAFs*) were widely expressed in various tissues. In the Ia clade, the five homologs of *AtARF2* were highly expressed in various tissues, such as leaves, roots, stems, and flowers, which is similar to those of *Arabidopsis* [58]. The four homologs of *AtARF9* were greatly expressed in roots; the three homologs of *AtARF1* had high expression levels in roots, stems, and cotyledon; however, the two homologs of *AtARF11* had divergent expression patterns, where one gene was highly expressed in seeds, seed coating, and cotyledon, and the others exhibited low expression in all tissues.

In subfamily II, all five *BnARFs* had a similar expression pattern, and most of them were highly expressed in roots and stems. A few genes were partially expressed in flowers. In subfamily III, the homologs of *AtARF6/8* were mainly expressed in flowers; the homologs of *AtARF19* were highly expressed in roots and stems; the homolog of *AtARF7* (*BnARF04*) was highly expressed in roots, stems, leaves, flowers, and silique, whereas the homologs of *AtARF5* were mainly expressed in roots and seed tissues in *B*. *napus*. In subfamily IV, the homologs of *AtARF17* were mainly expressed in roots, stems, and seed coating, and the homologs of *AtARF10*/*16* were mainly highly expressed in flowers and/or seed tissues. Taken together, the expression profile analysis showed that *BnARFs* may be involved in multiple processes throughout *B*. *napus* development, and that the functions of homologs in a given clade might be redundant as they share similar expression patterns.

### Expression analysis of *B*. *napus* ARFs under hormone induction

**Fig 7:**
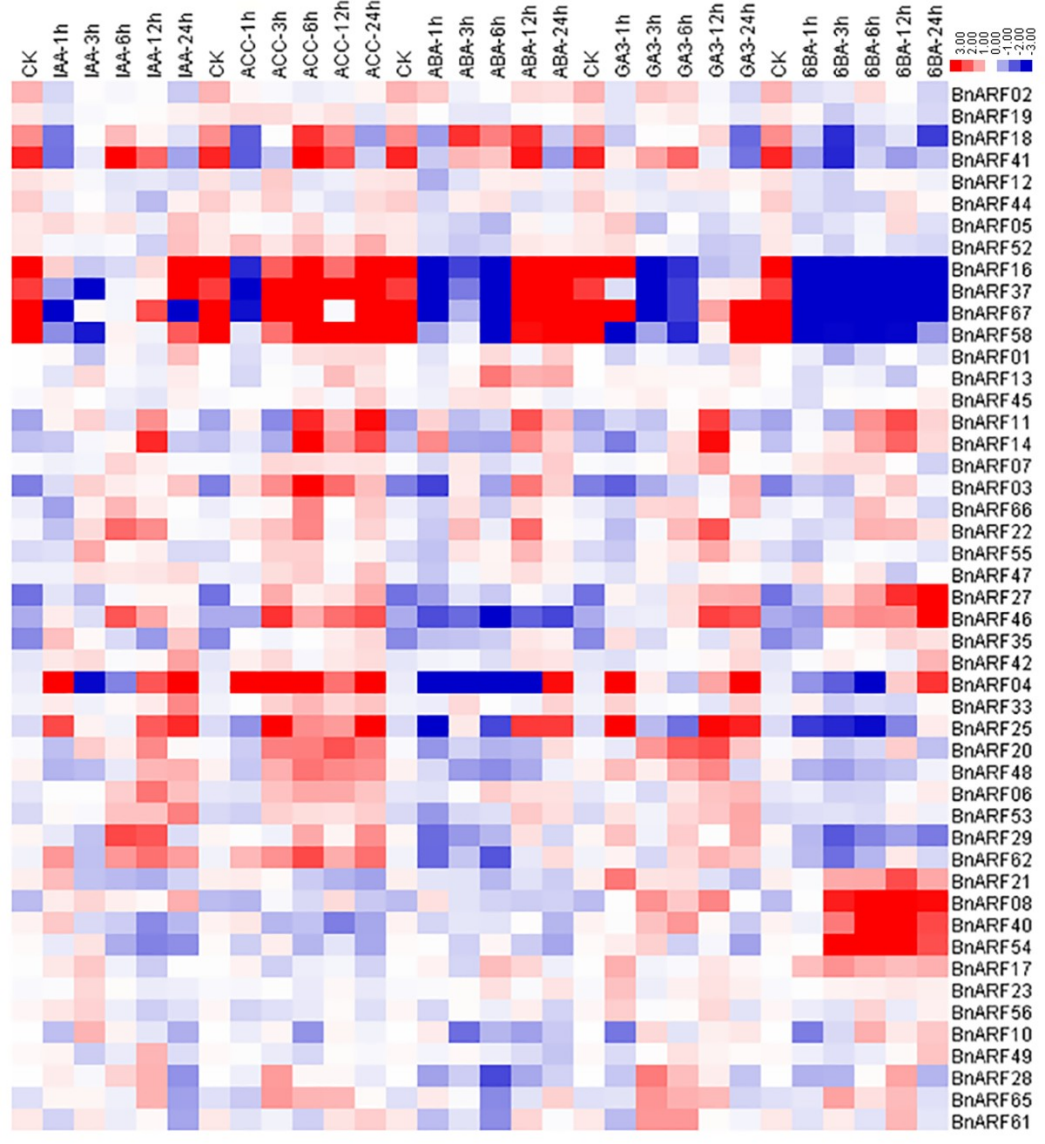
Expression profiles of *BnARFs* under five hormone induction in *B*. *napus* seedling roots by RNA-seq. IAA: indoleacetic acid, ACC: 1-aminocyclopropanecarboxylic acid, ABA: abscisic acid, GA3: gibberellin acid 3, 6-BA: cytokinin. The “1”, “3”, “6”, “12”, and “24” represent hours after treatment. Genes with no or weak expression levels (FPKM <1) are not included. The color bar at the top represents log_2_ expression values: green represents low expression, black represents mean expression level, and red represents high expression.

As ARF genes are well-known for their roles in the auxin-response process, which is important for root development, we also explored the expression patterns of *BnARFs* in *B*. *napus* roots under five exogenous hormone treatments (IAA, ACC, ABA, GA3, and 6-BA) (Fig 7).

For the IAA treatment, eight genes from subfamily I (*BnARF42*, *BnARF11*, and *BnARF14*), III (*BnARF22*, *BnARF55*, and *BnARF04*), and IV (*BnARF53* and *BnARF06*) were up-regulated while one gene in subfamily I (*BnARF02*) was suppressed. For the GA3 treatment, five genes from subfamily I (*BnARF11* and *BnARF14*) and III (*BnARF22*, *BnARF55*, and *BnARF23*) were up-regulated, and no genes were suppressed. For the ABA treatment, four genes from subfamily I (*BnARF11* and *BnARF14*) and III (*BnARF22* and *BnARF13*) were up-regulated, and one gene from subfamily III (*BnARF12*) was suppressed. For the ACC treatment, three genes from subfamily I (*BnARF11* and *BnARF14*) and III (*BnARF04*) were up-regulated, and one gene from subfamily IV (*BnARF41*) was suppressed. For the 6-BA treatment, seven genes from subfamily I (*BnARF42*, *BnARF11*, and *BnARF14*), II (*BnARF08*), and III (*BnARF27*, *BnARF40*, and *BnARF54*) were up-regulated, and two genes from subfamily I (*BnARF02*) and IV (*BnARF01*) were suppressed.

Overall, two genes (*BnARF11* and *BnARF14*) were commonly up-regulated under these five hormone treatments. Two genes (*BnARF22* and *BnARF55*) were up-regulated by IAA and GA3 treatments (*BnARF22* was also up-regulated by the ABA treatment). One gene (*BnARF42*) was up-regulated by IAA and 6-BA, and one gene (*BnARF02*) was suppressed by IAA and 6-BA. It has been previously reported that the expression of *AtARF4*, *AtARF16*, and *AtARF19* genes could be up-regulated by IAA treatment in *Arabidopsis* [59, 60]. Accordingly, in *B*. *napus*, we found that the homologs of *AtARF19* (*BnARF22* and *BnARF55*) and *AtARF16* (*BnARF06* and *BnARF53*) were also up-regulated by the IAA treatment (Fig 7). Taken together, our results suggest that the genes in subfamily III are more susceptible to hormones than those in other subfamilies, and that these *BnARFs* were more sensitive to exogenous IAA and 6-BA treatments.

**Fig 8:**
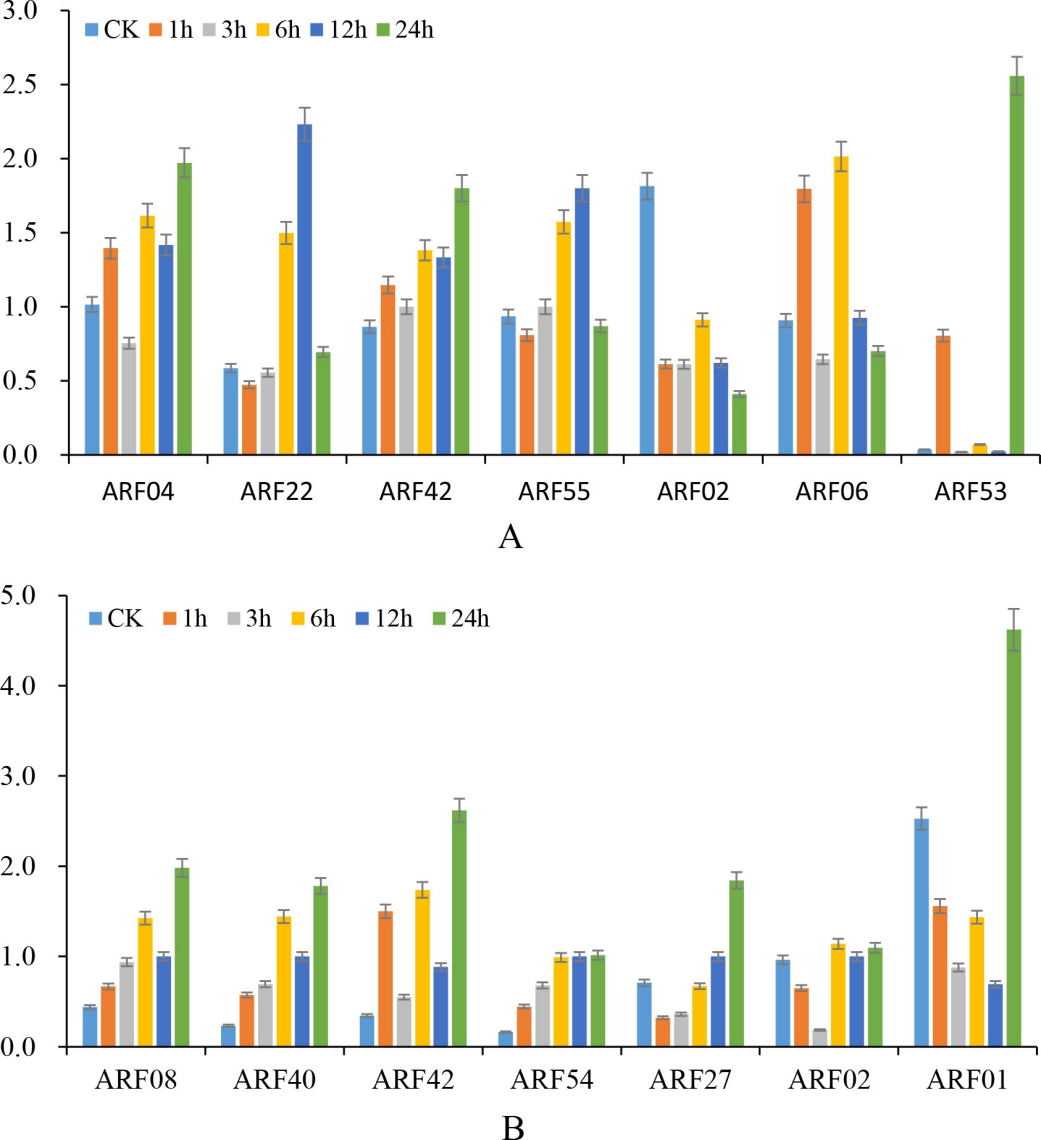
Expression profiles of *BnARFs* under IAA and 6-BA treatments. A and B: The transcript levels of candidate *BnARFs* in seedling roots by qRT-PCR under IAA and 6-BA treatments. CK = control (0 h). Data are mean ± standard deviation of three replicates.

To further verify the results of the RNA-seq analyses, twelve *BnARFs* (*BnARF01*, *BnARF02*, *BnARF04*, *BnARF05*, *BnARF06*, *BnARF08*, *BnARF22*, *BnARF27*, *BnARF55*, *BnARF40*, *BnARF42*, and *BnARF54*) with complete coding sequences were selected to investigate their responses to IAA and 6-BA treatments using qRT-PCR. The results were similar to those of our RNA-seq analysis and the candidates were evidently differentially regulated by IAA and 6-BA treatments (Fig 8). Under IAA treatment, six genes (*BnARF04*, *BnARF22*, *BnARF42*, *BnARF55*, *BnARF06*, and *BnARF53*) were upregulated, among which the expressions of *BnARF22* at 6 and 12 h, *BnARF42* at 24 h, *BnARF06* at 6 h, and *BnARF53* at 1 and 24 h were more than two-fold upregulated compared to 0 h. One gene (*BnARF02*) was significantly downregulated under the IAA treatment. Under the 6-BA treatment, five genes (*BnARF08*, *BnARF40*, *BnARF42*, *BnARF54*, and *BnARF53*) were upregulated, two genes (*BnARF01* and *BnARF02*) were downregulated, and one gene (*BnARF27*) was downregulated at 1 h and then upregulated at 24 h. Moreover, as shown in Fig 6, up to 10 genes of the 12 candidate *BnARFs* (*BnARF01*, *BnARF02*, *BnARF04*, *BnARF08*, *BnARF22*, *BnARF27*, *BnARF40*, *BnARF42*, *BnARF54*, and *BnARF55*) were highly expressed in root tissues; only two genes (*BnARF06* and *BnARF53*) have no detectable expression levels in roots but were obviously upregulated by IAA and 6-BA inductions in roots. Together, these results identify these genes as candidates for further study on the potential roles of *BnARFs* in hormone response in *B*. *napus* root.

## Discussion

### ARF gene increase, functional redundancy, and differentiation in *B*. *napus*

In the present study, we systematically predicted and identified 67 ARF members in the *B*. *napus* genome, which represents the largest ARF gene family identified in plants to date. Considering that *B*. *napus* originated through a natural doubling of chromosomes following the hybridization of *B*. *rapa* and *B*. *oleracea*, and that *Brassicaceae* species experienced a common whole genome triplication (WGT) event, it was expected that the 23 *Arabidopsis* ARF genes may be expanded to ~70 and ~140 genes in *B*. *rapa*/*B*. *oleracea* and *B*. *napus* genomes, respectively. However, only 29, 33, and 67 genes were found in these three species, respectively. In *B*. *napus*, the number of genes in the A_n_- and C_n_- subgenomes were almost the same as that in their diploid progenitors *B*. *rapa* and *B*. *oleracea*. These findings indicate that there many ARF genes were lost after the *Brassicaceae* WGT, with 58% of ARF genes in *B*. *oleracea* and 52% of ARF genes in *B*. *rapa* being lost. However, most of the duplicated ARF genes were retained after the WGD event. Thus, the WGD is the main source of gene expansion in *B*. *napus*. Together, the different retention ratios between these two large expansion events (WGD and WGT) may be because the WGT event occurred ~9–15 Ma [61,62] or 28 Ma [63–65], whereas the WGD event occurred only ~7500 years ago, which may be too short for the gene loss experienced by their older relatives. The WGD event duplicated many genes in *B*. *napus*. It would be interesting to further explore the fates of these duplicates, such as gene loss [66], neofunctionalization [67], and subfunctionalization [66]. Accordingly, many *BnARFs* have very similar expression patterns, such as *BnARF07/66*, *BnARF19/47*, *BnARF27/46*, *BnARF12/44*, etc. (S7 Table).

Then, we calculated the sequence similarity and sequence identity of B3, full protein, DNA, and the promoter sequence, and analyzed the expression pattern of 65 synteny-pairs in *B*. *napus*. We found that all synteny-pairs had highly similar B3 domains (the sequence similarity was > 90%), while only three synteny-pairs (*BnARF14/45*, *BnARF14/56*, and *BnARF14/23*) had low similarity in all sequences, which may due to the large fragment deletion in *BnARF14*. The sequence similarity of the full length of the proteins was a little reduced, and 51 synteny-pairs (78%) shared over 80% sequence similarity. However, the DNA sequence similarity was obviously reduced, and there were 27 synteny-pairs (42%) sharing over 80% sequence similarity. The promoter sequences of the synteny-pairs had the lowest sequence similarity with only one pair (*BnARF19/47*) having sequence similarity >80% (91.5%). These results indicate that neofunctionalization of duplicated genes may occur in nucleic acid sequences firstly, especially in the promoter regions.

Pearson correlation coefficient analysis of the expression patterns of 65 synteny-pairs in *B*. *napus* (S7 Table) showed that 19 of the 65 (29%) pairs had almost the same expression pattern, 18 pairs (28%) had similar expression patterns, and 21 (32%) pairs had different expression patterns. Seven pairs were not expressed in *B*. *napus*. Accordingly, we can speculate that most synteny-pair genes are functionally redundant, and that some synteny-pair genes have undergone sequence differentiation leading to differentiation of expression. These results suggest that the differences in expression patterns may result from neofunctionalization of duplicates during evolution.

### Activation and repression roles of ARF genes in the auxin signaling pathway

Auxin is a key regulator of virtually every aspect of plant growth and development from embryogenesis to senescence by controlling gene expression via the ARF gene family. In this process, ARF proteins represent the core of auxin signaling [68]. The key components of this pathway are the transport inhibitor resistant 1/auxin signaling f-box (TIR1/AFB) F-box proteins, the transcriptional co-regulators auxin/indole-3-acetic acid (Aux/IAA), and ARFs. Among them, the roles of ARF genes are divided into two types: transcriptional activator and transcriptional repressor. The activity of ARF activators is enabled through the auxin-dependent degradation of the Aux/IAA repressors [68]. In the absence of auxin, Aux/IAA proteins and ARF activators form heterodimers to prevent ARF-mediated transcription [4,17,69]. In the presence of auxin, auxin binds to the pocket in the TIR1/AFB protein, and the affinity for Aux/IAA proteins increases [70]. Aux/IAA proteins are subsequently ubiquitinated [71] and degraded in the 26S proteasome [72,73]. Therefore, ARF activity is depressed and numerous auxin-mediated transcriptional changes occur [19,68, 71].

Although ARF activators conform to the established ARF signaling pathway, the role of ARF repressors remains less clear [74]. It is still not clear how the ARF repressors regulate gene repression and how other transcription factors and signaling proteins interact with ARF proteins. Consequently, functions of ARF repressors have been proposed, such as squelching the transcriptional activity of ARF activators either by heterodimerizing with them or through competition for DNA binding sites [17]. Alternatively, some ARF repressors might homodimerize or heterodimerize and recruit co-repressor such as TPL/TPR (TOPLESS and related) proteins [75]. Little information is available to distinguish between these alternatives, as the full catalogue of potential *in planta* ARF dimers/oligomers is not available. However, the co-expression of ARF activators and repressors in the same tissue [17] is probably functionally relevant for auxin-dependent transcription. It was proposed that ARF repressors are involved in auxin signaling in both a competitive and cooperative fashion with ARF activators [76]. The activator/repressor categorization correlates with the divisions in subfamilies (Fig 2).

In *B*. *napus*, those ARFs identified as activators belong to subfamily III, while ARFs associated with subfamilies I, II, and IV are repressors. According to our prediction, subfamily III only interacts with Aux/IAA and ARF proteins, while the other subfamilies can interact with Aux/IAA proteins and many other types of proteins, such as ARF, BIN2, TPR1/2/3, and SWI3A/3B/3C. These results demonstrate the different mechanisms of ARF activators and repressors where the activators regulate auxin response by interacting with Aux/IAA. Conversely, the repressors act in a competitive role in the same pathway, and the activate or suppress functions of these ARFs on target genes creates an equilibrium that is critical in during auxin reactions.

## Conclusions

A total of 67 ARF genes were identified in the *B*. *napus* genome, which were further classified to four subfamilies (I–IV) with conserved intron patterns and protein motifs in each subfamily or clade. The full-length protein structures of *BnARFs* were highly conserved in each subfamily or clade. The evolutionary relationship between *B*. *napus*, *B*. *rapa*, *B*. *oleracea*, and *Arabidopsis* demonstrated that WGD and segmental duplication can be attributed to the rapid expansion of ARF genes in *B*. *napus*, and that this may be independent from tandem duplication. The duplicates derived from *B*. *rapa* tend to be retained in *B*. *napus* compared to those from *B*. *oleracea*. Also, most of the synteny-pairs in *B*. *napus* have similar expression patterns indicating functional redundancy. Meanwhile, there are some synteny-pairs that underwent expression divergence and are likely to occur primarily in the promoter regions. The results of the present study provide valuable information regarding the expansion and evolution of the ARF gene family in *B*. *napus* following allopolyploidy.

## Supporting information

S1 Fig Amino acid composition at the C-terminal region of the MR of the transcriptional repressors and activators in *B. napus*. (PDF)

S2 Fig Classification of the cis-elements in *BnARF* promoters. (PDF)

S1 Table List of the primers used for the Real-time PCR analyses. (XLSX)

S2 Table ARF gene family in *Arabidopsis, B. oleracea*, and *B. rapa* genomes. (XLSX)

S3 Table The orthologous-pairs between *AtARF–BnARF, BrARF–BnARF* and *BoARF–BnARF* genes. (XLSX)

S4 Table Duplications of ARF genes in *B. napus, B. rapa*, and *B. oleracea*. (XLSX)

S5 Table Potential miRNA targets of *B. napus* ARF genes. (XLSX)

S6 Table List of the interaction proteins in *Arabidopsis* and *B. napus*. (XLSX)

S7 Table Homology and expression analysis of 65 *BnARF* synteny-pairs. (XLSX)

## References

[1] Guilfoyle TJ, Hagen G. Auxin response factors. Curr Opin Plant Biol. 2007; 10(5): 453460 10.1016/j.pbi.2007.08.014 17900969

[2] Tiwari SB, Hagen G, Guilfoyle TJ. The roles of auxin response factor domains in auxin-responsive transcription. Plant Cell. 2003; 15: 533–543. 10.1105/tpc.008417 12566590 PMC141219

[3] Boer DR, Freire-Rios A, van den Berg WA, Saaki T, Manfield IW, Kepinski S, et al. Structural basis for DNA binding specificity by the auxin-dependent ARF transcription factors. Cell. 2014; 156(3):577–589. 10.1016/j.cell.2013.12.027 24485461

[4] Ulmasov T, Hagen G, Guilfoyle TJ. Dimerization and DNA binding of auxin response factors. Plant J. 1999; 19: 309–319. 10476078 10.1046/j.1365-313x.1999.00538.x

[5] Ulmasov T, Hagen G, Guilfoyle TJ. Activation and repression of transcription by auxin-response factors. Proc Natl Acad Sci USA. 1999; 96(10): 5844–5849. 10318972 10.1073/pnas.96.10.5844 PMC21948

[6] Liscum E, Reed JW. Genetios of Aux/IAA and ARF action in plant growth and development. Plant Mol. Biol. 2002; 49(3–4): 387–400. 10.1023/A:1015255030047 12036262

[7] Wang S, Hagen G, Guilfoyle TJ. ARF-Aux/IAA interactions through domain III/IV are not strictly required for auxin-responsive gene expression. Plant Signal Behav. 2013; 8(6):e24526 10.4161/psb.24526 23603958 PMC3909085

[8] Ulmasov T, Murfett J, Hagen G, Guilfoyle TJ. Aux/IAA proteins repress expression of reporter genes containing natural and highly active synthetic auxin response elements. Plant Cell. 1997; 9(11):1963–1971. 10.1105/tpc.9.11.1963 9401121 PMC157050

[9] Hagen G, Guilfoyle T. Auxin-responsive gene expression: genes, promoters and regulatory factors. Plant Mol Biol. 2002; 49(3–4):373–385. 12036261

[10] Okushima Y, Overvoorde PJ, Arima K, Alonso JM, Chan A, Chang C, et al Functional genomic analysis of the AUXIN RESPONSE FACTOR gene family members in Arabidopsis thaliana: unique and overlapping functions of ARF7 and ARF19. Plant Cell. 2005; 17(2):444–463. 10.1105/tpc.104.028316 15659631 PMC548818

[11] Abel S, Theologis A. Early genes and auxin action. Plant Physiol. 1996; 111(1):9–17. . 8685277 10.1104/pp.111.1.9 PMC157808

[12] Quint M, Gray WM. Auxin signaling. Curr Opin Plant Biol. 2006; 9(5):448–453. 10.1016/j.pbi.2006.07.006 16877027 PMC2424235

[13] Woodward AW, Bartel B. Auxin: regulation, action, and interaction. Ann Bot. 2005; 95:707–735. 10.1093/aob/mci083 15749753 PMC4246732

[14] Wang B, Xue JS, Yu YH, Liu SQ, Zhang JX, Yao XZ, et al Fine regulation of ARF17 for anther development and pollen formation. BMC Plant Biol. 2017; 17(1): 243 10.1186/s12870-017-1185-1 29258431 PMC5735505

[15] Liu Z, Miao L, Huo R, Song X, Johnson C, Kong L, et al ARF2-ARF4 and ARF5 are Essential for Female and Male Gametophyte Development in Arabidopsis. Plant Cell Physiol. 2018; 59(1): 179–189. 10.1093/pcp/pcx174 29145642

[16] Zhang X, Yan F, Tang Y, Yuan Y, Deng W, Li Z, et al Auxin Response Gene SlARF3 Plays Multiple Roles in Tomato Development and is Involved in the Formation of Epidermal Cells and Trichomes. Plant Cell Physiol. 2015; 56(11):2110–2124. 10.1093/pcp/pcv136 26412778

[17] Vernoux T, Brunoud G, Farcot E, Morin V, Van den Daele H, Legrand J, et al The auxin signalling network translates dynamic input into robust patterning at the shoot apex. Molecular Systems Biology. 2011; 7:508 10.1038/msb.2011.39 21734647 PMC3167386

[18] Han M, Park Y, Kim I, Kim EH, Yu TK, Rhee S, et al Structural basis for the auxin-induced transcriptional regulation by Aux/IAA17. Proc Natl Acad Sci U S A. 2014; 111(52):18613–18618. 10.1073/pnas.1419525112 25512488 PMC4284525

[19] Korasick DA, Westfall CS, Lee SG, Nanao MH, Dumas R, Hagen G, et al Molecular basis for AUXIN RESPONSE FACTOR protein interaction and the control of auxin response repression. Proc Natl Acad Sci U S A. 2014; 111(14):5427–5432. 10.1073/pnas.1400074111 24706860 PMC3986151

[20] Nanao MH, Vinos-Poyo T, Brunoud G, Thévenon E, Mazzoleni M, Mast D, et al Structural basis for oligomerization of auxin transcriptional regulators. Nat Commun. 2014; 5:3617 10.1038/ncomms4617 24710426

[21] Zouine M, Fu Y, Chateigner-Boutin AL, Mila I, Frasse P, Wang H, et al Characterization of the Tomato ARF Gene Family Uncovers a Multi-Levels Post-Transcriptional Regulation Including Alternative Splicing. PLoS One. 2014; 9(1):e84203 10.1371/journal.pone.0084203 24427281 PMC3888382

[22] Singh VK, Rajkumar MS, Garg R, Jain M. Genome-wide identification and co-expression network analysis provide insights into the roles of auxin response factor gene family in chickpea. Scientific Reports. 2017; 7(1):10895 10.1038/s41598-017-11327-5 28883480 PMC5589731

[23] Liu J, Hua W, Hu Z, Yang H, Zhang L, Li R et al Natural variation in ARF18 gene simultaneously affects seed weight and silique length in polyploid rapeseed. Proc Natl Acad Sci U S A. 2015 9 15;112(37):E5123–32. 10.1073/pnas.1502160112 26324896 PMC4577148

[24] Berardini TZ, Reiser L, Li D, Mezheritsky Y, Muller R, Strait E, et al The Arabidopsis Information Resource: Making and Mining the ‘Gold Standard’ Annotated Reference Plant Genome. Genesis. 2015; 53(8):474–485. 10.1002/dvg.22877 26201819 PMC4545719

[25] Thompson JD, Gibson TJ, Plewniak F, Jeanmougin F, Higgins DG. The CLUSTAL_X windows interface: flexible strategies for multiple sequence alignment aided by quality analysis tools. Nucleic Acids Res. 1997; 25(24):4876–4882. 10.1093/nar/25.24.4876 9396791 PMC147148

[26] Artimo P, Jonnalagedda M, Arnold K, Baratin D, Csardi G, de Castro E, et al ExPASy: SIB bioinformatics resource portal. Nucleic acids research. 2012; 40: W597–603. 10.1093/nar/gks400 22661580 PMC3394269

[27] Chou KC, Shen HB. Cell-PLoc: a package of Web servers for predicting subcellular localization of proteins in various organisms. Nat Protoc. 2008; 3(2):153–162. 10.1038/nprot.2007.494 18274516

[28] Voorrips RE. MapChart: software for the graphical presentation of linkage maps and QTLs. J Hered. 2002; 93(1):77–78. . 12011185 10.1093/jhered/93.1.77

[29] Bjellqvist B.; Hughes G.J.; Pasquali C.; Paquet N.; Ravier F.; Sanchez J.C.; Frutiger S.; Hochstrasser D. The focusing positions of polypeptides in immobilized pH gradients can be predicted from their amino acid sequences. Electrophoresis 1993, 14, 1023–1031. 10.1002/elps.11501401163 . 8125050

[30] Tamura K, Peterson D, Peterson N, Stecher G, Nei M, Kumar S. MEGA5: molecular evolutionary genetics analysis using maximum likelihood, evolutionary distance, and maximum parsimony methods. Mol Biol Evol. 2011; 28(10):2731–2739. 10.1093/molbev/msr121 21546353 PMC3203626

[31] Bailey TL, Boden M, Buske FA, Frith M, Grant CE, Clementi L, et al MEME SUITE: tools for motif discovery and searching. Nucleic acids research. 2009; 37:W202–208. 10.1093/nar/gkp335 19458158 PMC2703892

[32] Szklarczyk D, Morris JH, Cook H, Kuhn M, Wyder S, Simonovic M, et al The STRING database in 2017: quality-controlled protein-protein association networks, made broadly accessible. Nucleic Acids Res. 2017; 45(D1):D362–D368. 10.1093/nar/gkw937 27924014 PMC5210637

[33] Shannon P, Markiel A, Ozier O, Baliga NS, Wang JT, Ramage D, et al Cytoscape: A Software Environment for Integrated Models of Biomolecular Interaction Networks. Genome Research. 2003; 13(11):2498–2504. 10.1101/gr.1239303 14597658 PMC403769

[34] Hu B, Jin J, Guo AY, Zhang H, Luo J, Gao G. GSDS 2.0: an upgraded gene feature visualization server. Bioinformatics. 2015; 31:1296–1297. 10.1093/bioinformatics/btu817 25504850 PMC4393523

[35] Dai X, Zhao PX. psRNATarget: A Plant Small RNA Target Analysis Server. Nucleic Acids Res. 2011; 39:W155–159. 10.1093/nar/gkr319 21622958 PMC3125753

[36] Goodstein DM, Shu S, Howson R, Neupane R, Hayes RD, Fazo J, et al Phytozome: a comparative platform for green plant genomics. Nucleic Acids Res. 2012; 40:D1178–1186. 10.1093/nar/gkr944 22110026 PMC3245001

[37] Saldanha AJ. Java Treeview—extensible visualization of microarray data. Bioinformatics. 2004; 20(17):3246–3248. 10.1093/bioinformatics/bth349 . 15180930

[38] Du H, Ran F, Dong HL, Wen J, Li JN, Liang Z. Genome-Wide Analysis, Classification, Evolution, and Expression Analysis of the Cytochrome P450 93 Family in Land Plants. PLOS ONE. 2016; 10.1371/journal.pone.0165020 27760179 PMC5070762

[39] Die JV, Gil J, Millan T. Genome-wide identification of the auxin response factor gene family in Cicer arietinum. BMC Genomics. 2018; 19(1):301 10.1186/s12864-018-4695-9 29703137 PMC5921756

[40] Zhou X, Wu X, Li T, Jia M, Liu X, Zou Y, et al Identification, characterization, and expression analysis of auxin response factor (ARF) gene family in *Brachypodium distachyon*. Funct Integr Genomics. 2018; 10.1007/s10142-018-0622-z 29926224

[41] Wang D, Pei K, Fu Y, Sun Z, Li S, Liu H, et al Genome-wide analysis of theauxin response factors (ARF) gene family in rice (Oryza sativa). Gene. 2007; 394(1–2):13–24. 10.1016/j.gene.2007.01.006 17408882

[42] Xu Z, Ji A, Song J, Chen S. Genome-wide analysis of auxin response factor gene family members in medicinal model plant *Salvia miltiorrhiza*. Biol Open. 2016; 5(6):848–857. 10.1242/bio.017178 27230647 PMC4920185

[43] Yu H, Soler M, Mila I, San Clemente H, Savelli B, Dunand C et al Genome-Wide Characterization and Expression Profiling of the AUXIN RESPONSE FACTOR (ARF) Gene Family in Eucalyptus grandis. PLoS One. 2014; 9(9):e108906 10.1371/journal.pone.0108906 25269088 PMC4182523

[44] Xing H, Pudake RN, Guo G, Xing G, Hu Z, Zhang Y, et al Genome-wide identification and expression profiling of auxin response factor (ARF) gene family in maize. BMC Genomics. 2011; 12:178 10.1186/1471-2164-12-178 21473768 PMC3082248

[45] Kumar R, Tyagi AK, Sharma AK. Genome-wide analysis of auxin response factor (ARF) gene family from tomato and analysis of their role in flower and fruit development. Mol Genet Genomics. 2011; 285(3):245–260. 10.1007/s00438-011-0602-7 21290147

[46] Yu H, Soler M, Mila I, San Clemente H, Savelli B, Dunand C, et al Genome-wide characterization and expression profiling of the AUXIN RESPONSE FACTOR (ARF) gene family in *Eucalyptus grandis*. PLoS One. 2014; 9(9):e108906 10.1371/journal.pone.0108906 eCollection 2014 25269088 PMC4182523

[47] Wan S, Li W, Zhu Y, Liu Z, Huang W, Zhan J. Genome-wide identification, characterization and expression analysis of the auxin response factor gene family in *Vitis vinifera*. Plant Cell Rep. 2014; 33(8):1365–1375. 10.1007/s00299-014-1622-7 24792421

[48] Higgins EE, Clarke WE, Howell EC, Armstrong SJ, Parkin IAP. Detecting de novo homoeologous recombination events in cultivated *Brassica napus* using a genome-wide SNP array. G3 (Bethesda). 2018; 8(8):2673–2683. 10.1534/g3.118.200118 29907649 PMC6071606

[49] Hardtke CS, Ckurshumova W, Vidaurre DP, Singh SA, Stamatiou G, Tiwari SB, et al Overlapping and non-redundant functions of the Arabidopsis auxin response factors MONOPTEROS and NONPHOTOTROPIC HYPOCOTYL 4. Development. 2004;131(5):1089–1100. 10.1242/dev.00925 14973283

[50] Wang S, Tiwari SB, Hagen G, Guilfoyle TJ. AUXIN RESPONSE FACTOR7 restores the expression of auxin-responsive genes in mutant Arabidopsis leaf mesophyll protoplasts. Plant Cell. 2005; 17(7):1979–1993. 10.1105/tpc.105.031096 15923351 PMC1167546

[51] Wilmoth JC, Wang S, Tiwari SB, Joshi AD, Hagen G, Guilfoyle TJ, et al NPH4/ARF7 and ARF19 promote leaf expansion and auxin-induced lateral root formation. Plant J. 2005; 43(1):118–130. 10.1111/j.1365-313X.2005.02432.x 15960621

[52] Du H, Liang Z, Zhao S, Nan MG, Tran LS, Lu K, et al The Evolutionary History of R2R3-MYB Proteins Across 50 Eukaryotes: New Insights Into Subfamily Classification and Expansion. Sci Rep. 2015; 5:11037 10.1038/srep11037 26047035 PMC4603784

[53] Wu MF, Tian Q, Reed JW. Arabidopsis microRNA167 controls patterns of ARF6 and ARF8 expression, and regulates both female and male reproduction. Development. 2006; 133:4211–4218. 10.1242/dev.02602 17021043

[54] Wang JW, Wang LJ, Mao YB, Cai WJ, Xue HW, Chen XY. Control of root cap formation by microRNA-targeted auxin response factors in Arabidopsis. Plant Cell. 2005; 17(8):2204–2216. 10.1105/tpc.105.033076 16006581 PMC1182483

[55] Colón-Carmona A, Chen DL, Yeh KC, Abel S. Aux/IAA proteins are phosphorylated by phytochrome in vitro. Plant Physiol. 2000; 124(4):1728–1738. 10.1104/pp.124.4.1728 11115889 PMC59870

[56] Li J, Dai X, Zhao Y. A Role for Auxin Response Factor 19 in auxin and ethylene signaling in Arabidopsis. Plant Physiol. 2006; 140(3): 899–908. 10.1104/pp.105.070987 16461383 PMC1400570

[57] Vert G, Walcher CL, Chory J, Nemhauser JL. Integration of auxin and brassinosteroid pathways by Auxin Response Factor 2. Proc Natl Acad Sci USA. 2008; 105(28):9829–9834. 10.1073/pnas.0803996105 18599455 PMC2474533

[58] Ulmasov T, Hagen G, Guilfoyle TJ. ARF1, a transcription factor that binds to auxin response elements. Science. 1997; 276(5320):1865–1868. 9188533 10.1126/science.276.5320.1865

[59] Ellis CM, Nagpal P, Young JC, Hagen G, Guilfoyle TJ, Reed JW. AUXIN RESPONSE FACTOR1 and AUXIN RESPONSE FACTOR2 regulate senescence and floral organ abscission in *Arabidopsis thaliana*. Development. 2005; 132(20):4563–4574. 10.1242/dev.02012 16176952

[60] Li J, Dai X, Zhao Y. A Role for Auxin Response Factor 19 in Auxin and Ethylene Signaling in Arabidopsis. Plant Physiol. 2006;140(3):899–908. 10.1104/pp.105.070987 16461383 PMC1400570

[61] Wang X, Wang H, Wang J, Sun R, Wu J, Liu S, et al The genome of the mesopolyploid crop species *Brassica rapa*. Nat Genet. 2011; 43(10):1035–1039. 10.1038/ng.919 21873998

[62] Beilstein MA, Nagalingum NS, Clements MD, Manchester SR, Mathews S. Dated molecular phylogenies indicate a Miocene origin for Arabidopsis thaliana. Proc Natl Acad Sci U S A. 2010; 107(43):18724–18728. 10.1073/pnas.0909766107 20921408 PMC2973009

[63] Lukens L, Quijada PA, Udall JA, Pires JC, Schranz E, Osborn TC et al Genome redundancy and plasticity within ancient and recent Brassica crop species. Biol J Linnean Soc. 2004; 82: 665–674. 10.1111/j.1095-8312.2004.00352.x

[64] Lysak MA, Koch MA, Pecinka A, Schubert I. Chromosome triplication found across the tribe Brassiceae. Genome Res. 2005; 15(4): 516–525. 10.1101/gr.3531105 15781573 PMC1074366

[65] Arias T, Beilstein MA, Tang M, McKain MR, Pires JC. Diversification times among *Brassica* (*Brassicaceae*) crops suggest hybrid formation after 20 million years of divergence. Am J Bot. 2014; 101(1):86–91. 10.3732/ajb.1300312 24388963

[66] Piya S, Shrestha SK, Binder B, Stewart CNJr, Hewezi T. Protein–protein interaction and gene co-expression maps of ARFs and Aux/IAAs in Arabidopsis. Front Plant Sci. 2014; 5:744 10.3389/fpls.2014.00744 25566309 PMC4274898

[67] Blanc G, Wolfe KH. Widespread paleopolyploidy in model plant species inferred from age distributions of duplicate genes. Plant Cell. 2004; 16(7):1667–1678. 10.1105/tpc.021345 15208399 PMC514152

[68] Paponov IA, Paponov M, Teale W, Menges M, Chakrabortee S, Murray J.A. Comprehensive transcriptome analysis of auxin responses in Arabidopsis. Mol Plant. 2008; 1(2):321–337. 10.1093/mp/ssm021 19825543

[69] Chapman EJ, Estelle M. Mechanism of auxin-regulated gene expression in plants. Annu Rev Genet. 2009; 43:265–285. 10.1146/annurev-genet-102108-134148 19686081

[70] Szemenyei H, Hannon M, Long JA. TOPLESS mediates auxin-dependent transcriptional repression during Arabidopsis embryogenesis. Science. 2008; 319(5868):1384–1386. 10.1126/science.1151461 18258861

[71] Tan X, Calderon-Villalobos LI, Sharon M, Zheng C, Robinson CV, Estelle M, et al Mechanism of auxin perception by the TIR1 ubiquitin ligase. Nature. 2007; 446(7136):640–645. 10.1038/nature05731 17410169

[72] Maraschin FS, Memelink J, Offringa R. Auxin-induced, SCFTIR1 -mediated poly-ubiquitination marks AUX/IAA proteins for degradation. Plant J. 2009; 59(1):100–109. 10.1111/j.1365-313X.2009.03854.x 19309453

[73] Gray WM, Kepinski S, Rouse D, Leyser O, Estelle M. Auxin regulates SCF TIR1–dependent degradation of AUX/IAA proteins. Nature. 2001; 414(6861):271–276. 10.1038/35104500 11713520

[74] Zenser N, Ellsmore A, Leasure C, Callis J. Auxin modulates the degradation rate of Aux/IAA proteins. Proc Natl Acad Sci U S A. 2001; 98(20):11795–11800. 10.1073/pnas.211312798 11573012 PMC58810

[75] Roosjen M, Paque S, Weijers D. Auxin response factors: output control in auxin biology. J Exp Bot. 2018; 69(2):179–188. 10.1093/jxb/erx237 28992135

[76] Causier B, Ashworth M, Guo W, Davies B. The TOPLESS interactome: a framework for gene repression in Arabidopsis. Plant Physiol. 2012;158(1):423–438. 10.1104/pp.111.186999 22065421 PMC3252085

