# Supplementary material for: The auxin response factor gene family in allopolyploid Brassica napus
Source: PLoS One. 2019 Apr 8;14(4):e0214885. doi: 10.1371/journal.pone.0214885 (PMC6453480; doi:10.1371/journal.pone.0214885)
Supplement: S1 Fig — (PDF) [file pone.0214885.s001.pdf]

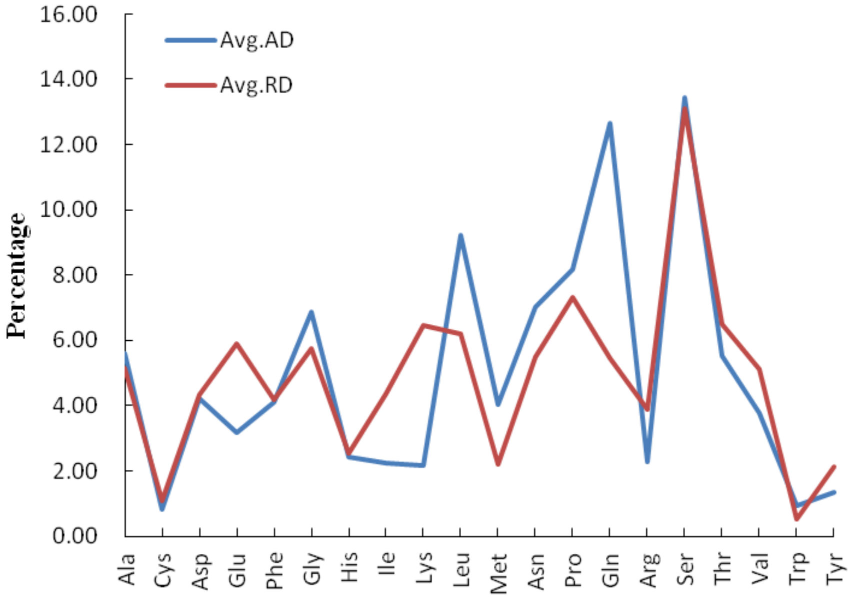

S1 Fig. Amino acid composition at the C-terminal region of the MR of the transcriptional repressors and activators in *B. napus*. The red and blue lines indicate the percentages of the residues at each site between ARF transcriptional activators (Avg.AD) and transcriptional repressors (Avg.RD) in *B. napus*.
