# Supplementary material for: The auxin response factor gene family in allopolyploid Brassica napus
Source: PLoS One. 2019 Apr 8;14(4):e0214885. doi: 10.1371/journal.pone.0214885 (PMC6453480; doi:10.1371/journal.pone.0214885)
Supplement: S2 Fig — (PDF) [file pone.0214885.s002.pdf]

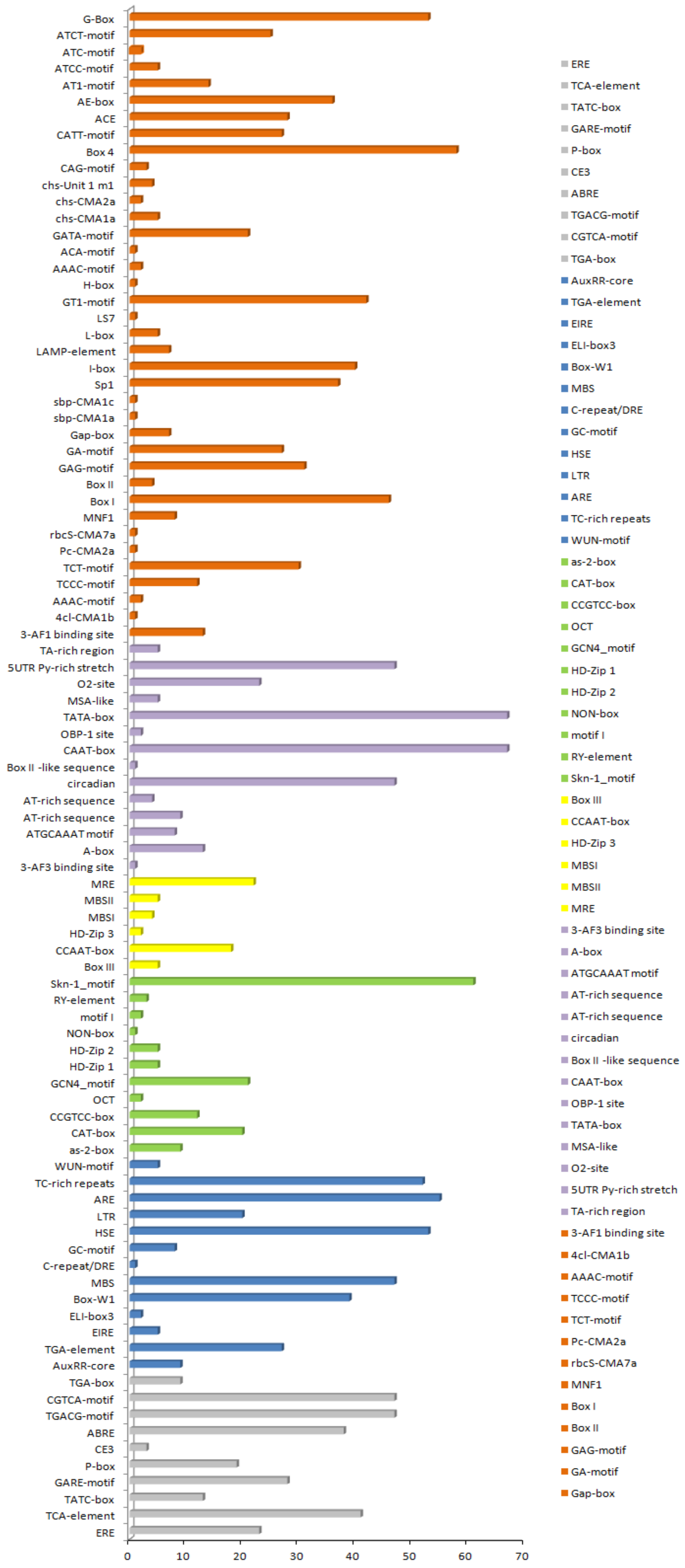
G-Box
ATCT-motif
ATC-motif
ATCC-motif
AT1-motif
AE-box
ACE
CATT-motif
Box 4
CAG-motif
chs-Unit 1 m1
chs-CMA2a
chs-CMA1a
GATA-motif
ACA-motif
AAAC-motif
H-box
GT1-motif
LS7
L-box
LAMP-element
I-box
Sp1
sbp-CMA1c
sbp-CMA1a
Gap-box
GA-motif
GAG-motif
Box II
Box I
MNF1
rbcS-CMA7a
Pc-CMA2a
TCT-motif
TCCC-motif
AAAC-motif
4cl-CMA1b
3-AF1 binding site
TA-rich region
5UTR Py-rich stretch
O2-site
MSA-like
TATA-box
OBP-1 site
CAAT-box
Box II -like sequence
circadian
AT-rich sequence
AT-rich sequence
ATGCAAAT motif
A-box
3-AF3 binding site
MRE
MBSII
MBSI
HD-Zip 3
CCAAT-box
Box III
Skn-1_motif
RY-element
motif I
NON-box
HD-Zip 2
HD-Zip 1
GCN4_motif
OCT
CCGTCC-box
CAT-box
as-2-box
WUN-motif
TC-rich repeats
ARE
LTR
HSE
GC-motif
C-repeat/DRE
MBS
Box-W1
ELI-box3
EIRE
TGA-element
AuxRR-core
TGA-box
CGTCA-motif
TGACG-motif
ABRE
CE3
P-box
GARE-motif
TATC-box
TCA-element
ERE
0
10
20
30
40
50
60
70
ERE
TCA-element
TATC-box
GARE-motif
P-box
CE3
ABRE
TGACG-motif
CGTCA-motif
TGA-box
AuxRR-core
TGA-element
EIRE
ELI-box3
Box-W1
MBS
C-repeat/DRE
GC-motif
HSE
LTR
ARE
TC-rich repeats
WUN-motif
as-2-box
CAT-box
CCGTCC-box
OCT
GCN4_motif
HD-Zip 1
HD-Zip 2
NON-box
motif I
RY-element
Skn-1_motif
Box III
CCAAT-box
HD-Zip 3
MBSI
MBSII
MRE
3-AF3 binding site
A-box
ATGCAAAT motif
AT-rich sequence
AT-rich sequence
circadian
Box II -like sequence
CAAT-box
OBP-1 site
TATA-box
MSA-like
O2-site
5UTR Py-rich stretch
TA-rich region
3-AF1 binding site
4cl-CMA1b
AAAC-motif
TCCC-motif
TCT-motif
Pc-CMA2a
rbcS-CMA7a
MNF1
Box I
Box II
GAG-motif
GA-motif
Gap-box

S2 Fig. Classification of the cis-elements in *BnARF* promoters. Cis-acting components are divided into six categories in different color. Gray: hormone response elements; blue: biological stress response elements; green: developmental and differentiation-related cis-acting elements; yellow: protein binding sites; purple: basic cis-elements; brown: light signaling perception cis-elements.
